# “Jumping Jack”: Genomic Microsatellites Underscore the Distinctiveness of Closely Related *Pseudoperonospora cubensis* and *Pseudoperonospora humuli* and Provide New Insights Into Their Evolutionary Past

**DOI:** 10.3389/fmicb.2021.686759

**Published:** 2021-07-14

**Authors:** Marcin Nowicki, Denita Hadziabdic, Robert N. Trigiano, Sarah L. Boggess, Loukas Kanetis, Phillip A. Wadl, Peter S. Ojiambo, Marc A. Cubeta, Otmar Spring, Marco Thines, Fabian Runge, Brian E. Scheffler

**Affiliations:** ^1^Department of Entomology and Plant Pathology, University of Tennessee, Knoxville, Knoxville, TN, United States; ^2^Department of Agricultural Sciences, Biotechnology & Food Science, Cyprus University of Technology, Limassol, Cyprus; ^3^USDA-ARS, Vegetable Research, Charleston, SC, United States; ^4^Department of Entomology and Plant Pathology, Center for Integrated Fungal Research, North Carolina State University, Raleigh, NC, United States; ^5^Institute of Botany 210, University of Hohenheim, Stuttgart, Germany; ^6^Department of Biological Sciences, Biodiversity and Climate Research Centre (BiK-F), Senckenberg Gesellschaft fuer Naturforschung and Evolution and Diversity, Institute of Ecology, Goethe University, Frankfurt am Main, Germany; ^7^IDENTXX GmbH, Stuttgart, Germany; ^8^U.S. Department of Agriculture, Agricultural Research Service, Stoneville, MS, United States

**Keywords:** oomycete, obligate pathogens, downy mildew, speciation, evolution, genotyping, host specificity

## Abstract

Downy mildews caused by obligate biotrophic oomycetes result in severe crop losses worldwide. Among these pathogens, *Pseudoperonospora cubensis* and *P. humuli*, two closely related oomycetes, adversely affect cucurbits and hop, respectively. Discordant hypotheses concerning their taxonomic relationships have been proposed based on host–pathogen interactions and specificity evidence and gene sequences of a few individuals, but population genetics evidence supporting these scenarios is missing. Furthermore, nuclear and mitochondrial regions of both pathogens have been analyzed using microsatellites and phylogenetically informative molecular markers, but extensive comparative population genetics research has not been done. Here, we genotyped 138 current and historical herbarium specimens of those two taxa using microsatellites (SSRs). Our goals were to assess genetic diversity and spatial distribution, to infer the evolutionary history of *P. cubensis* and *P. humuli*, and to visualize genome-scale organizational relationship between both pathogens. High genetic diversity, modest gene flow, and presence of population structure, particularly in *P. cubensis*, were observed. When tested for cross-amplification, 20 out of 27 *P. cubensis*-derived gSSRs cross-amplified DNA of *P. humuli* individuals, but few amplified DNA of downy mildew pathogens from related genera. Collectively, our analyses provided a definite argument for the hypothesis that both pathogens are distinct species, and suggested further speciation in the *P. cubensis* complex.

## Introduction

Downy mildews caused by a group of obligate oomycete pathogens are responsible for severe crop losses in numerous hosts worldwide (Spring et al., 2018). Among the pathogens causing various downy mildews, *Pseudopernospora cubensis* (Berk. & M.A. Curt.) Rostov. and *P. humuli* (Miyabe & Takah.) G.W. Wilson that affect cucurbits and hop (*Humulus lupulus* L.), respectively, are particularly important owing to their considerable economic (Gent and Ocamb, 2009; Savory et al., 2011), financial, and environmental impacts related to crop protection (Reuveni et al., 1980; Gent et al., 2008). These impacts were exacerbated after the 2004 re-emergence of cucumber-invasive lineage of *P. cubensis* and its rapid spread in North America (Colucci, 2008; Holmes et al., 2015; Ojiambo et al., 2015; Spring et al., 2018).

Taxonomic descriptions of *P. cubensis* and *P. humuli* have been revised several times based on the emerging phytopathological, epidemiological, and molecular evidence (Choi et al., 2005; Savory et al., 2011). The 2005 controversy called for a reduction of both taxa to a single species based on morphological measurements and lack of polymorphisms in their ITS sequences (Choi et al., 2005). This claim has since been disregarded by the inclusion of additional nuclear and mitochondrial sequences (Mitchell et al., 2011; Runge and Thines, 2012), detection of cryptic species in *P. cubensis* (Runge et al., 2011), transcriptomic comparisons (Summers et al., 2015b), and robust phytopathological studies (Mitchell et al., 2011; Runge and Thines, 2012). An additional important argument against conspecificity is the difference in the sexual reproduction of both pathogens: heterothallism in *P. cubensis* (Cohen and Rubin, 2012) and homothallism in *P. humuli* (Gent et al., 2017). Several studies have discussed the critical need for a comparative molecular population-genetics approach to investigate and help settle this issue (Sarris et al., 2009; Mitchell et al., 2011; Runge and Thines, 2012; Kitner et al., 2015; Summers et al., 2015b).

To date, only three studies (Chee et al., 2006; Gent et al., 2019; Kitner et al., 2021) have analyzed the *P. humuli* diversity using a population genetics approach. Chee et al. (2006) suggested differences in the population structures and reproduction modes due to climate in Oregon and Washington, using the RAPD markers for genotyping on a local scale. Gent et al. (2019) used genotyping-by-sequencing of the 2010 and 2011 pathogen populations from those same U.S. states to discover limited genetic diversity in *P. humuli*, in line with repeated inbreeding in this homothallic oomycete. Kitner et al. (2021) tested cross-amplification of short sequence repeats (SSRs) developed for *P. cubensis* (Wallace and Quesada-Ocampo, 2017) on the *P. humuli* collected from Central Europe around the Alps. They also developed five SSRs that were more polymorphic than the other tested, suggesting higher genetic uniformity of *P. humuli* in Europe than in North America and prompting their hypothesis of the pathogen being introduced to Europe (Kitner et al., 2021). Comparatively, several molecular approaches were used for population biology of *P. cubensis* (Quesada-Ocampo et al., 2012; Polat et al., 2014; Kitner et al., 2015; Naegele et al., 2016; Thomas et al., 2017a; Wallace and Quesada-Ocampo, 2017). Intersimple sequence repeat (ISSR) and sequence-related amplified polymorphism (SRAP) markers used for the analyses of *P. cubensis* from Israel, Turkey, and Czech Republic indicated high diversity both among and within the regions, with the samples from Israel forming a distinct clade (Polat et al., 2014). This study suggested possible sexual reproduction of the pathogen in Israel, constituting the source of inoculum for the greater region (Polat et al., 2014). The global population of *P. cubensis* from five host species was analyzed by comparing the sequences of five nuclear and two mitochondrial regions (Quesada-Ocampo et al., 2012). As a result, the population structures by location and by host were evident, informing of pathogen variability and suggesting the development of customized control measures (Quesada-Ocampo et al., 2012). Sequencing of 12 molecular regions from the *P. cubensis* collected across the Czech Republic indicated temporal changes in pathogen, coinciding with its global resurgence (Kitner et al., 2015). Diversity of the 2011 *P. cubensis* collection from Michigan and Ontario was analyzed using nine SSRs and showed high population structure in space and variation in time, as well as differences by the host species (Naegele et al., 2016). Recently, transcriptomes of *P. cubensis* and *Hyaloperonospora arabidopsidis* (Gäum.) Göker, Riethm., Voglmayr, Weiß & Oberw. were screened for SSRs that were later confirmed in collections of *P. cubensis* and *P. humuli*. Eleven of such markers were polymorphic and showed that genetic diversity was higher in *P. humuli* than in *P. cubensis* (Wallace and Quesada-Ocampo, 2017). Finally, Wallace et al. (2020) genotyped the collection of 385 isolates of *P. cubensis* from six commercial and four wild cucurbits from North Carolina, using 10 SSRs. That study supported the existence of genetically distinct host-adapted clades in that pathogen, at the cucurbit species level (Quesada-Ocampo et al., 2012; Wallace et al., 2020).

Among the molecular tools commonly used by the modern population genetics, SSRs are still widely used, as they occur frequently in the genomic (gDNA; gSSRs), genic (RNA; eSSRs), and cytoplasmic parts of the genome (mitochondrial DNA and chloroplast DNA if present). They are multiallelic in character and exhibit relatively high mutation rates that allow the assessment of species diversity and inferences of their evolutionary past (Rubinsztein et al., 1995; Gobbin et al., 2003; Bhargava and Fuentes, 2010). This can help inform the related control measures for pathogens, pests, or other undesirable invasive species (Cornuet et al., 2014; Goss et al., 2014; Wu et al., 2016; Kim et al., 2019). Of note, our group previously reported gSSRs for *P. cubensis* (Kanetis et al., 2009). The gSSRs reported therein and other gSSRs developed at that time were used extensively in the present work.

In this study, we utilized previously developed gSSRs based on the gDNA of *P. cubensis* (Kanetis et al., 2009) to genotype a collection of gDNA from historical (herbarium specimens) and post-re-emergence samples of both *P. cubensis* and *P. humuli* pathogens. We hypothesized that both taxa did not belong to a single species and diverged recently. Our study goal was to test that hypothesis based on the following specific research objectives: (i) to identify distinctive features that separate the two taxa using population genetics tools—basic diversity analyses, (ii) to analyze the population structure in both taxa using Bayesian and multivariate tools, (iii) to infer the evolutionary history of both pathogens using the approximate Bayesian clustering and approximate computation, and (iv) to visualize the genome-scale organizational relationship between both pathogens utilizing comparative genomics analyses of the available draft genomes.

## Materials and Methods

### Pathogen Samples

A total of 138 samples of *P. cubensis* (*n* = 77) and *P. humuli* (*n* = 61) were included in this study (Table 1 and Supplementary Table 1). Samples of *P. cubensis* were obtained from at least eight various cucurbit hosts (two specimens were of unknown hosts), whereas the *P. humuli* samples were all obtained from hop (Supplementary Table 1). Samples of *P. cubensis* were subdivided into the following groups based on their geographic origin and time of collection: historical North American (“PcUSAh”; dated 1887 to 1935; *n* = 18), historical Old World (“PcEh”; Europe and South-East Asia; 1890 to 1955; *n* = 9), current North American (“PcUSAc”; North Carolina; 2008; *n* = 24), and current European (“PcEc”; 1998 to 2014; *n* = 28). Samples of *P. humuli* of the current European (“PhEc”; 2008 to 2010; *n* = 56) and historical origin (“PhEh; *n* = 3; Czech Republic 1940; Poland 1957; Argentina 1980) were also included. This constituted the “six-subpopulations” dataset used in the subsequent analyses. Historical samples were obtained by limited destructive sampling of downy mildew-symptomatic specimens from the following herbaria: New York Botanical Garden (NY), the Herbarium of the Plant Diseases Division of New Zealand (PDD), the Farlow Herbarium of the Harvard University (FH), the Mycological Herbarium of the Korea University (KUS-F), and the Herbarium of the University of Hohenheim (HOH) with respective permissions granted by our requests; “fresh” material was collected by Dr. Fabian Runge. gDNA of herbarium and “fresh” material was extracted using the Analytikjena Plant Kit as previously described (Telle and Thines, 2008).

**TABLE 1:** Population genetics indices for the *Pseudoperonospora cubensis* and *P. humuli* collection genotyped using the genomic simple sequence repeats (gSSRs).

Population^*a*^	*n* ^*c*^	%amp	N_*A*_	*N*_*AE*_	*A*_*R*_	*P*_*A*_	*λ*	*H*_*O*_	*μ H*_*E*_	*H*	*F*_*I*_	$\bar{\text{r}}$ _*d*_
**Six-subpopulations dataset**												
PcEc^*b*^	9	96.8	21	2.33	2.10	3	0.89	0.20	0.52	2.20	0.62***	0.50***
PcEh	9	95.2	29	4.01	2.60	2	0.89	0.18	0.69	2.20	0.76***	0.14**
PcUSAc	13	93.4	18	2.05	1.86	1	0.92	0.31	0.42	2.56	0.27**	0.10**
PcUSAh	13	95.6	27	2.76	2.27	4	0.92	0.24	0.57	2.56	0.59***	0.21***
PhEc	30	90.0	23	2.03	1.97	1	0.97	0.04	0.49	3.40	0.92***	0.10**
PhEh	3	85.7	9	1.28	1.27	0	0.67	0.00	0.15	1.10	1.00*	-0.50^*ns*^
Overall	77	92.8	127	3.80	2.67	11	0.99	0.15	0.71	4.31	0.79***	0.16***
**Species dataset—population-wise indices**												
Pc	49	95.6	47	3.57	5.92	28	0.977	0.23	0.70	3.76	0.67***	0.22***
Ph	29	89.2	20	1.72	2.94	1	0.969	0.04	0.39	3.47	0.90***	0.10***
Overall	78	93.2	67	3.57	5.85	29	0.987	0.16	0.69	4.32	0.76***	0.16***

*^*a*^Indices for each subpopulation and the overall values calculated for the whole dataset are presented.*

*^*b*^Subpopulation codes denote the following: *P. cubensis* (Pc) historical North American (dated 1887 to 1935) (PcUSAh), historical Old World (Europe and South-East Asia; 1890 to 1955) (PcEh), current North American (North Carolina; 2008) (PcUSAc), and current European (1998 to 2014) (PcEc). Samples of *P. humuli* (Ph): current European (2008 to 2010) (PhEc) and historical origin (Czech Republic 1940; Poland 1957; Argentina 1980) (PhEh).*

^*c*^*n*, number of samples per subpopulation, after clone correction; %amp, % of samples amplified using seven gSRRs; *N*_*A*_, number of alleles detected per subpopulation; *N*_*AE*_, number of effective alleles per subpopulation; *A*_*R*_, allelic richness, rarefied automatically; *P*_*A*_, number of private alleles detected per subpopulation; *λ*, Simpson’s index of diversity (Simpson, 1949); *H*_*O*_, observed heterozygosity (Hardy and Vekemans, 2002); *μH*_*E*_, heterozygosity expected under Hardy–Weinberg equilibrium or gene diversity corrected for sample size (Hardy and Vekemans, 2002; Nielsen et al., 2003); *F*_*I*_, individual inbreeding coefficient with *p*-value after 9,999 randomization of gene copies among individuals (Hardy and Vekemans, 2002); significance levels: ns, not significant; *p < 0.05, **p < 0.01, ***p < 0.0001; and $\bar{r}$_*d*_: standardized index of association (Agapow and Burt, 2001).

### Development of gSSR Markers, Cross-Amplification of gSSRs, and Genotyping Conditions

SSR markers for *P. cubensis* were generated using enriched genomic libraries (Wang et al., 2007; Kanetis et al., 2009; Trigiano et al., 2012) on DNA sample NC01. Genomic locations of the gSSRs were established in the available *P. cubensis* and *P. humuli* draft genomes (Savory et al., 2012; Rahman et al., 2019) using NCBI BLAST Genomes service (Altschul et al., 1990). All redundant hits found with comparable reliability (coverage%, identity%, E-value), were recorded (Supplementary Table 2). In this study, 27 gSSRs were used to test cross-amplification across gDNA samples from samples of *P. cubensis* (*n* = 17), *P. humuli* (*n* = 6), *P. humuli* ex *Humulus japonicus* (*n* = 1), *P. celtidis* (*n* = 3), *P. urticae* (*n* = 3), *P. cannabina* (*n* = 2), *Bremia lactucae* (*n* = 2), and *Benua kellermannii* (*n* = 1) (Supplementary Table 2).

For population studies, the DNA samples of *P. cubensis* and *P. humuli* were genotyped with the following seven gSSRs—PC004, PC008, PC017, PC018, PC020, PC021, and PC027 (Table 2 and Supplementary Table 1)—using previously established modified protocols (Kanetis et al., 2009). Our modifications included touchdown cycling program (Korbie and Mattick, 2008) to avoid spurious amplification products: 94°C for 3 min; 15 cycles of 94°C for 40 s, 40 s initially at 63°C and subsequently decreasing 0.5°C per cycle, and 72°C for 30 s. The next 20 cycles consisted of 94°C for 40 s, 55°C for 40 s, and 72°C for 30 s, ending with 72°C for 4 min. The PCR mixture contained the following in the total volume of 10 μl: 2 ng of gDNA, 2 mM MgCl_2_, 2.5 mM dNTPs, 1 μM each primer, 1 μl DMSO, 1 × PCR buffer, and 1 U AmpliTaq Gold (Thermo Fisher Scientific, Inc., Waltham, MA, United States). Amplification products were visualized using the QIAxcel Advanced system (Qiagen, Valencia, CA, United States) according to the described conditions (Wang et al., 2009; Trigiano et al., 2012) and sized using the 15/600-bp alignment marker and 25- to 500-bp size marker (Qiagen). Loci that did not amplify after three consecutive reactions were considered missing data.

**TABLE 2:** Locus-wise indices for the *Pseudoperonospora cubensis* and *P. humuli* collection genotyped using the genomic simple sequence repeat markers (gSSRs).

Locus^*a*^	%amp	Size (bp)	*N*_*A*_	*N*_*AE*_	*A*_*R*_	*H*_*O*_	*μ H*_*E*_	*N*_*m*_	*D*_*est*_	*R*_*IS*_	*R*_*ST*_
**Six-subpopulations dataset**											
PC004	83.1	138–144	5	2.93	2.44	0.00	0.66	0.30	0.66	1.00***	0.49**
PC008	90.9	126–144	8	2.75	2.44	0.03	0.64	0.17	0.47	0.99***	0.51***
PC017	98.7	196–228	10	3.87	2.78	0.53	0.74	0.50	0.40	-0.54***	0.33***
PC018	98.7	150–154	5	2.86	2.43	0.00	0.65	0.40	0.35	1.00***	0.33***
PC020	97.4	170–208	6	3.09	2.57	0.03	0.68	1.14	0.29	1.00***	0.33***
PC021	97.4	182–196	10	6.68	3.21	0.03	0.85	0.37	0.79	0.83***	0.71***
PC027	83.1	196–219	5	4.41	2.84	0.47	0.77	0.41	0.57	-0.08^*ns*^	0.33***
Overall	92.8		7.00	3.8	2.67	0.15	0.71	0.41	0.52	0.71***	0.39***
**Species dataset—locus-wise indices**											
PC004	83.3	138–144	5	2.90	4.30	0.00	0.66	0.59	0.59	1.00***	0.18**
PC008	91	126–144	7	2.49	5.61	0.01	0.60	1.41	0.41	1.00***	0.27***
PC017	98.7	196–228	10	3.83	7.78	0.58	0.74	0.55	0.57	-0.49***	0.39***
PC018	98.7	150–154	5	2.68	4.37	0.00	0.63	3.16	0.18	1.00***	0.18**
PC020	97.4	170–208	6	2.49	5.39	0.01	0.60	4.50	0.14	1.00***	0.16*
PC021	97.4	182–196	10	6.23	8.56	0.01	0.84	1.03	0.91	0.92***	0.77***
PC027	85.9	196–219	5	4.35	4.92	0.52	0.77	1.23	0.64	-0.05^*ns*^	0.29***
Overall	93.2		6.86	3.57	5.85	0.16	0.69	1.78	0.48	0.74***	0.29***

*Indices for each gSSR and the overall values calculated for the whole dataset are presented.*

^*a*^For each locus (PC#), shown are the following indices: %amp, % of samples amplified using a given gSRRs; size (bp), size range (bp) of the detected alleles; *N*_*A*_, number of alleles detected per subpopulation; *N*_*AE*_, number of effective alleles per subpopulation; *A*_*R*_, allelic richness, rarefied automatically; *H*_*O*_, observed heterozygosity (Hardy and Vekemans, 2002); *μH*_*E*_, heterozygosity expected under Hardy–Weinberg equilibrium or gene diversity corrected for sample size (Hardy and Vekemans, 2002; Nielsen et al., 2003); *N*_*m*_, estimated relative gene flow; *D*_*est*_, differentiation index (Jost, 2008; Kamvar et al., 2014); *R*_*IS*_, homozygote excess/deficiency, or inbreeding coefficient; *R*_*ST*_, allele fixation index (Slatkin, 1995; Michalakis and Excoffier, 1996; Hardy and Vekemans, 2002); significance after 9,999 randomization of gene copies among individuals: ^*ns*^, not significant; *p < 0.05, **p < 0.01, ***p < 0.0001.

### Hierarchical Indices and Genetic Diversity Indices

Raw allele lengths were binned into statistically distinct allelic classes using MS Excel macro FLEXIBIN (Amos et al., 2007). It utilized an algorithm to group the alleles into statistically uniform categories using the repeat length information. The binned dataset was then transformed to repeat number using the PGDSpider version 2.1.1.5 (Lischer and Excoffier, 2012), and that dataset was used for all subsequent analyses. The binned “six-subpopulations” dataset was then clone-corrected using *poppr* version 2.8.3 (Kamvar et al., 2014) in R version 3.6.1 (R Core Team, 2017), and the identical multilocus genotypes (MLGs) at the subpopulation level were removed to avoid biasing the analyses toward clonality. All subsequent analyses were completed using the clone-corrected data. The following standard indices were calculated: number of alleles and number of effective alleles observed at each locus or subpopulation (*N*_*A*_ and *N*_*AE*_; *SPAGeDi* version 1.5d; Hardy and Vekemans, 2002), allelic richness for populations or across tested loci (*A*_*R*_; rarefied as estimated automatically by *SPAGeDi*), private alleles (*P*_*A*_; calculated using GenAlEx version 6.5.1; Peakall and Smouse, 2012), observed heterozygosity (*H*_*O*_; by *SPAGeDi*), heterozygosity expected under Hardy–Weinberg equilibrium by *SPAGeDi* (*μH*_*E*_; gene diversity corrected for sample size; Nielsen et al., 2003) both in population- and locus-wise manner, *λ* (Simpson’s index of diversity; Simpson, 1949) calculated using *poppr*, *H* (Shannon–Wiener index of MLG diversity; Shannon, 1948) calculated using *poppr*, and $\bar{r}$_*d*_ [standardized index of association (Agapow and Burt, 2001) calculated using *poppr*]. Fixation indices statistics were calculated using *SPAGeDi*: *F*_*I*_ (individual inbreeding coefficient with *p*-value after 9,999 randomization of gene copies among individuals by *SPAGeDi*), inbreeding coefficients (*F*_*IS*_, *R*_*IS*_) and allele fixation indices by *SPAGeDi* (*F*_*ST*_, *R*_*ST*_, with significance after 9,999 randomization of gene copies among individuals (Slatkin, 1995; Michalakis and Excoffier, 1996), differentiation index (*D*_*est*_; *poppr*; Jost, 2008), and estimated gene flow (*N*_*m*_; GenAlEx). Genotype accumulation curve, departures from Hardy–Weinberg equilibrium, pairwise linkage disequilibrium, and population-wise Prevosti distance matrix (Prevosti, 1974) and its reticulation were calculated using packages *poppr*, *hierfstat* version 0.04-22 (Goudet, 2005), and *ape* version 5.3 (Paradis and Schliep, 2019) with default settings. Comparative data subdivision to separate the *P. cubensis* (*Pc*) and *P. humuli* (*Ph*) samples (“species” dataset) was also submitted to such analyses and the results are presented in Tables 1, 2.

The hierarchical partitioning of genetic variation of *P. cubensis* and *P. humuli* populations was analyzed using analysis of molecular variance (AMOVA) as implemented in *poppr*; *p*-values were obtained after 999 permutations. AMOVA partitions the total variance into hierarchical components (among groups, among individuals within groups, and within individuals). Three independent analyses were performed using (i) the “six-subpopulations” dataset, (ii) the “species” dataset, and (iii) the “host” dataset with subdivision by the affected host plants.

### Population Structure

Isolation-by-distance (IBD) was calculated using Mantel (*r*) and partial Mantel tests (*r′*; standardized by year of sampling), as implemented in R packages *MASS* version 7.3-51.4 (Ripley et al., 2013), *ade4* (Dray and Dufour, 2007), and *vegan* version 2.5-6 (Oksanen et al., 2013). The algorithm calculated the correlation of pairwise matrices of genetic and geographic distances among genotyped samples for either species. The same software calculated and plotted the correlograms of Mantel’s *r* across distance classes. Significance of the regression slope between genetic and logarithm of the geographical distances was assessed by 999 permutations.

Bayesian clustering method was applied to infer the number of the genetic clusters among *P. cubensis* and *P. humuli* samples using STRUCTURE version 2.3.3 (Pritchard et al., 2000; Hubisz et al., 2009). The analyses used 30 independent Monte Carlo Markov chains (MCMC) for each *k* value between one and six, with a burn-in period of 250,000 steps and 750,000 steps of the actual run, using an admixture model with correlated allele frequencies (assuming no prior information of subpopulation origin). Then, the Evanno method as implemented in the PopHelper version 1.0.10 was used to estimate the number of clusters using the *ad hoc* statistic Δ*k* (Evanno et al., 2005; Earl and vonHoldt, 2011; Francis, 2017). *Post-hoc* analysis using ObStruct version 1.0 (Gayevskiy et al., 2014) was used to analyze the overall fit of the predefined groups with the ancestry profiles inferred using STRUCTURE and to assess the contributions of each preattributed subpopulation and each inferred cluster. The program uses the *R*^2^ statistics that ranges from 0 (recent divergence or abundance of migration/admixture among subpopulations) to 1 (complete divergence and/or population structure). Three independent analyses used the “six-subpopulations,” “species,” and “host” datasets.

Model-free multivariate clustering analysis of the MLGs was carried out using the discriminant analysis of the principal components (DAPC; Jombart et al., 2010). The model was cross-checked and optimized using 1,000 permutations of the dataset across principal components used from 2 to 48. Pairwise population distance matrix (Prevosti, 1974) was reticulated and rooted using the OUTGROUP (Supplementary Table 1) with the neighbor-joining algorithm implemented in *poppr* using the “six-subpopulations” and “host” datasets.

### Demographic Analyses and Evolutionary History

To investigate for an occurrence of a recent population bottleneck, Bottleneck version 1.2.02 (Cornuet and Luikart, 1996) was utilized. The settings included two mutation modes that apply to SSRs [stepwise-mutation model (SMM) and two-phase mutational model (TPM) with 95% SMM]. Variance of the geometric distribution for TPM was set at 12. Both models used 10,000 iterations for all three basic tests: sign test, standardized differences test, and Wilcoxon sign rank test, to infer the mode shift indicative of recent demographic changes in the tested groups. Populations without a signature of a recent bottleneck followed the standard L-shaped distribution of allele frequencies as expected under mutation-drift equilibrium (Piry et al., 1999) based on the overall outcome of all three basic tests; detailed results of each test provide more insights for the observed bottleneck/population expansion. For this investigation, two independent analyses were performed (i) using the “three-subpopulations” dataset (North American *P. cubensis*, European *P. cubensis*, and European *P. humuli*) and (ii) the “species” dataset. The three-group split was used to increase the counts of individuals per subpopulation as required by Bottleneck and because the *P. cubensis* historical specimens showed alleles not detected in the respective current populations.

Evolutionary history of the species was investigated using the approximate Bayesian computation using the DIYABC version 2.1 (Cornuet et al., 2014). The focus of these analyses was on inferring the evolutionary relationships among both *Pseudoperonospora* species in relation to other downy mildew pathogens genotyped using the same seven gSSRs (Supplementary Tables 1, 2). The initial run established the limits of the input indices (population sizes, mutation rates) and used the entire dataset as one population, with the parameters stretched one order of magnitude beyond those defaulted by the program. The subsequent full DIYABC analyses were only run using the precalculated 95% confidence interval (95% CI) values for those indices using the “species” dataset (outgroup, *Pc*, *Ph*) and the “three-subpopulations” dataset (outgroup, *Pc* from Europe, *Pc* from United States, *Ph*). Detailed description of these analyses is presented in Supplementary File 1.

### Sequencing of Microsatellite Loci and Sequence Analyses

Multiple sequence comparison was performed to determine possible differences between both *Pseudoperonospora* species. Six gSSRs were amplified for *P. cubensis* (*n* = 17) and *P. humuli* samples (*n* = 5) using primer pairs for PC001, PC005, PC006, PC0012, PC018, and PC021 (Supplementary Table 2) according to the aforementioned protocol. PCR products were visualized on QIAxcel Advanced system as described above and ligated into pBluescript KS (−), transformed into *Escherichia coli* TOP10, and selected on LB medium supplemented with ampicillin and X-gal. The extracted plasmid DNA samples were submitted for Sanger sequencing using M13F and T3 primers (USDA-ARS, Stoneville, MS, United States) to generate consensus sequences for each locus and each sample, respectively. Owing to limited amounts of DNA in the pathogen collection, only a limited number of individuals were used for this analysis.

The consensus sequences for the six gSSR loci from the analyzed specimens were generated using Sequencher version 5.0.0 (Gene Codes Corporation, 2010), inspected, and manually corrected at the lower-quality termini (Supplementary Tables 2, 7). Sequence matrix for each locus was aligned separately using MAFFT version 7.0 (Katoh and Standley, 2013; Katoh et al., 2017) using default mode and settings and visualized using BioEdit version 7.0.5.3 (Hall, 1999). The sequence matrix was then manually concatenated in Microsoft Excel 2016. Identical sequences were removed before the downstream analysis, to avoid clonality bias. The concatenated sequence matrix was then analyzed using the maximum likelihood (ML) algorithm with thorough bootstrap, as implemented in raxmlGUI version 1.5 (Silvestro and Michalak, 2012; Stamatakis, 2014), with 100 repetitions and a bootstrap of 10,000. The RAxML used the ML algorithm with the molecular substitution matrix of GTR and the rapid hill-climbing mode. The results were visualized using FigTree version 1.4.3 (Rambaut, 2017).

To further assess the differences among both pathogens, the DIYABC analysis was implemented using the sequence matrix generated on six gSSRs amplified across 22 DNA samples from both species, as the aligned results presented consistent differences between the pathogens. Detailed description of this analytical algorithm is in Supplementary File 1.

### Comparative Genomics Analysis

To evaluate genome-scale differences between both pathogens, the publicly available draft genome assemblies of both *Pseudoperonospora* species were used (GenBank# for genome of *P. cubensis*: AHJF00000000.1; of *P. humuli*: NQFO00000000.1; Savory et al., 2012; Rahman et al., 2019). We used D-GENIES with *Minimap* version 2.0 (Cabanettes and Klopp, 2018) for the syntenic comparison of both *Pseudoperonospora* draft genomes. Additionally, physical variants between both draft genomic assemblies were also investigated with NUCMER (genomic DNA) and PROMER (six-frame translation) as implemented in MUMmer version 3.23 (Kurtz et al., 2004) and visualized using Assemblytics (Nattestad and Schatz, 2016), with the default settings except for “unique sequence length required” set at 1,000 for both genomic and translated genomic runs.

## Results

### SSR Cross-Amplification

Molecular similarity between *P. cubensis* and *P. humuli* was analyzed using the 27 gSSRs developed for the former species. From 27 selected gSSRs, 23 successfully amplified the *P. cubensis* samples, 20 gSSRs amplified the randomly selected representative samples of *P. humuli*, modest amplification to other *Pseudoperonospora* species was noted, and only four gSSRs (PC004, PC005, PC012, PC027) cross-amplified to related genera (Supplementary Tables 1, 2). Two of the latter SSRs shared similarity with critical metabolically active genes: PC005 with cytochrome c oxidase and PC012 with NADH dehydrogenase (Supplementary Table 2; BLAST refseq_rna, results not shown). Size ranges of the alleles detected using capillary electrophoresis were similar for both species of focus and well within the size range expected by the cloned fragments. Several gSSR sequences were not found in the inspected genomic assemblies despite cloning from the *P. cubensis* gDNA and successful amplification using DNA samples from both species (Supplementary Tables 1, 2).

### Population Genetics Analyses

#### Genetic Diversity, F-Statistics, and AMOVA

From the original collection of 138 DNA samples that were amplified using seven gSSRs (77 of *P. cubensis*, 61 of *P. humuli*), clone correction resulted in the removal of 62 clonal MLGs (Table 1) leaving 42 unique MLGs of *P. cubensis* and 34 of *P. humuli* for analyses. The seven gSSRs amplified an average of 93% (ranging from 83 to 99%) of the tested samples (Tables 1, 2 and Supplementary Table 1). About 21 alleles were detected per subpopulation (ranging from 9 to 27; Table 1). Private alleles were observed in all subpopulations except historical *P. humuli* from Europe. The overall allelic richness was 2.67 (1.27 to 2.60; Table 1). Both current subpopulations of *P. cubensis* from Europe and North America showed strong evidence for linkage disequilibrium, driving the overall linkage disequilibrium toward clonality ($\bar{r}$_*d*_ different from 0, *p* < 0.001; Table 1). The fixation indices suggested existence of population structure (overall *H*_*O*_ < *H*_*E*_; Tables 1, 2), with ample allele fixation (overall *R*_*ST*_ of 0.39) and very high genetic differentiation (*D*_*est*_ of 0.52). Inbreeding was also evident for subpopulations of both pathogens, with the overall *F*_*I*_ of 0.79 (Table 1). This, together with limited overall gene flow (*N*_*m*_ of 0.41 or 1.05; Table 2), suggests the clonal character of both pathogens. When data were partitioned based on two tested species, *P. cubensis* and *P. humuli*, our results showed similar patterns (Tables 1, 2).

The genotype accumulation curve indicated that the gSSRs performed well in accruing the diversity information from our collection, but roughly half of the MLGs were discarded due to clonality, and caused the lack of plateau in the curve. The remaining MLG pool was saturated with six gSSRs, irrespective of the dataset subdivision (Supplementary Figure 1). Deviations from Hardy–Weinberg equilibrium (HWE) were comparatively more frequent in the “species” dataset, but in the “six-subpopulations” dataset, PC027 and the smallest group PhEh showed the fewest HWE deviations (Supplementary Figure 2). This pattern was also reflected in the linkage disequilibrium $\bar{r}$_*d*_ values for both datasets (Table 1). Finally, the pairwise $\bar{r}$_*d*_ investigation among the seven used gSSRs indicated their low genetic linkage, implying the markers were dispersed across the *P. cubensis* genome (Supplementary Figure 3 and Supplementary Table 2). Permutation of the *R*_*ST*_ and comparison with the *F*_*ST*_ values (not shown) revealed that mutation was the major force in the observed differentiation, regardless of whether the analyses were run on the “six-subpopulations” or “species” dataset (*p* < 0.05 and *p* < 0.01, respectively; data not shown).

AMOVA indicated only minor differences in the variance partitioning depending on the dataset subdivision (Supplementary Table 3): change in partitioning the variance was evident among the subpopulations and among individuals. All three datasets congruently attributed the majority of variation to the individuals-within-subpopulations level, whereas the “six-subpopulations” dataset indicated a much stronger signal for separation among the subpopulations. This strongly implied the existence of population structure within the genotyped collection of *P. cubensis* and *P. humuli*, irrespective of the hierarchical organization of the datasets.

#### SSR Population Structure

Results of the isolation-by-distance tests (Mantel and partial Mantel) showed clear differences between *P. cubensis* and *P. humuli* groups. For the Mantel test, a significant correlation (*p* < 0.001) between geographical and genetic distances was evidenced, with about 13% of variance explained by geographical spread. When standardized by sampling year (partial Mantel test), this score was improved to about 14% of total variance (*p* < 0.001), indicating the minor temporal variability of *P. cubensis* over 127 years (1887 to 2014; Supplementary Table 1 and Supplementary Figure 4A). Furthermore, Mantel’s *r* scores were significant across the geographical classes, evidencing strong spatial impact on the population structure, with notable amplitude of Mantel score range and patches of (dis)similarity (Supplementary Figure 4B; vertical axis). In contrast, the results for *P. humuli* provided no such evidence, neither in raw data format, nor when standardized by the year of sampling (Supplementary Figures 4C,D), possibly reflecting the comparatively narrower geographic and temporal distribution in the collection.

**FIGURE 1:**
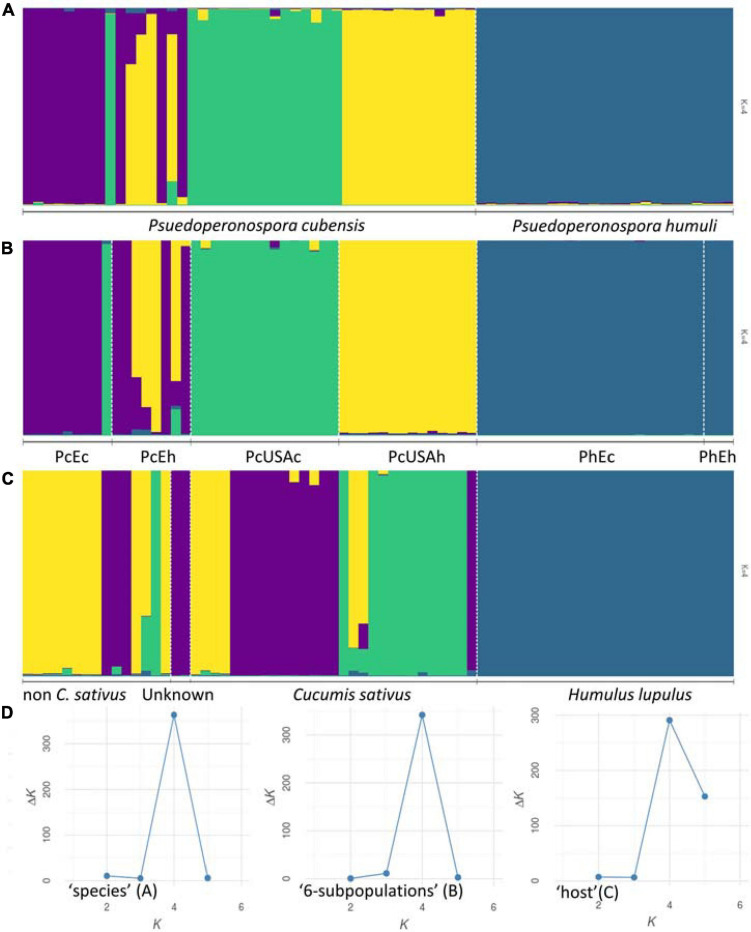
Population structure inferred using STRUCTURE. Genotyped dataset was split either by the preattributed species **(A)**, six groups that reflected the sample geographic origin and age [**(B)** *Pseudoperonospora cubensis*: historical North American (“PcUSAh”), historical Old World (“PcEh”; Europe and South-East Asia), current North American (“PcUSAc”), current European (“PcEc”); *P. humuli*: current European (“PhEc”) and historical origin (“PhEh”)], or the affected host plants **(C)**. The results were visualized using the most supported number of inferred genetic clusters **(D)** as per the Evanno method (Evanno et al., 2005).

Results of the Bayesian clustering analysis differed only slightly, depending on the dataset subdivision applied (i.e., “six-subpopulations,” “species,” or “host” datasets). This analysis provided congruent evidence for existence of four genetically distinct clusters within our collection (Figure 1). Samples of *P. humuli* were distinct from the *P. cubensis* group and formed a homogeneous separate cluster regardless of the input dataset. Comparatively, *P. cubensis* was comprised of two or three inferred clusters, depending on the dataset used. More specifically, the North American samples showed clear clustering distinction between the current and historical samples. Comparatively, the historical European samples were strongly admixed, implying an intensive gene flow between the continents (Figure 1B). The STRUCTURE results were then analyzed using ObStruct for additional insights of the accrued ancestry profiles (Supplementary Table 4). The highest *R*^2^ statistic value across the 30 used MCMC was calculated for the “six-subpopulations” dataset (0.91; *p* < 0.0001; Figure 1B), indicating strong divergence among the predefined subpopulations and population structure within the dataset. The lowest *R*^2^ was for the “species” dataset (0.43; *p* < 0.0001; Figure 1A), indicating their recent divergence. Only minor changes in the *R*^2^ values were observed for the “six-subpopulations” dataset when the preattributed populations or the inferred clusters were removed sequentially, which suggests that this dataset subdivision showed the best agreement with the inferred genetic clusters. In contrast, when analyzing the “host” dataset, removal of the *Cucumis sativus* subpopulation increased the *R*^2^ index value substantially, and removal of *H. lupulus* subpopulation decreased it the most. This indicated that those two groups carried the most variation and the strongest signal for population structure, respectively (Supplementary Table 4 and Figure 1C).

**FIGURE 2:**
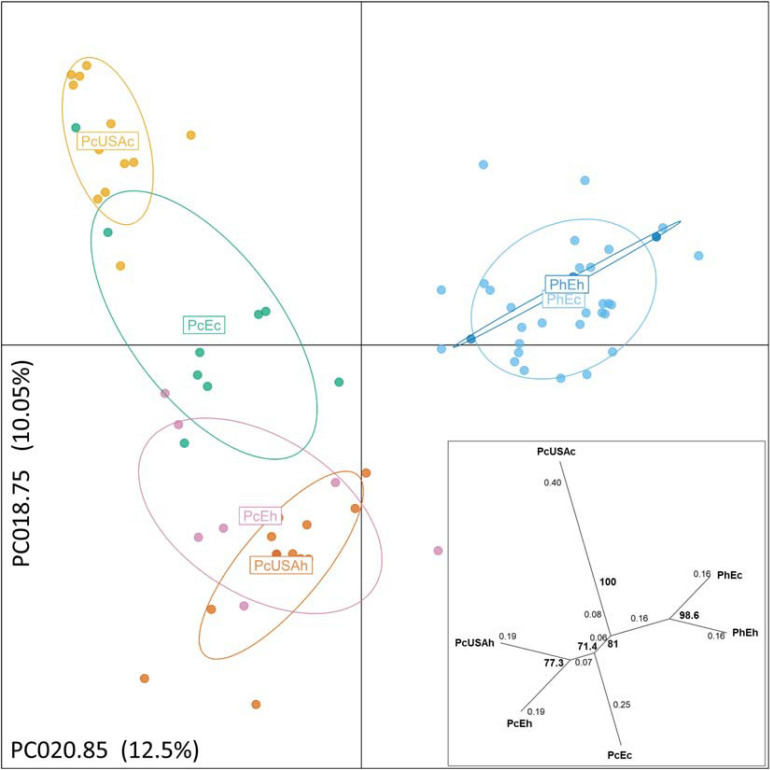
Discriminant analysis of principal components of the genotyped *Pseudoperonospora cubensis* and *P. humuli* separates the taxa and visualizes the substructure within. The pathogen groups are coded the same way as in Figure 1. The analysis was optimized by 1,000 permutations of the dataset across principal components analyzed from 2 to 48 toward the principal components used for the projection (PCA = 20). Two alleles contributing the most and used for the projection are identified along the respective axes, with % of total variance explained also indicated. Insert: pairwise population genetic distances (Prevosti, 1974) are reticulated with distances between each split indicated on the branches, and bootstrap support for the splits > 70% is indicated in bold, based on 1,000 permutations of the dataset.

Multivariate DAPC (Figure 2) provided additional support to the STRUCTURE results and resulted in similar patterns. In the “six-subpopulations” dataset, the taxa separation was evident. The current and historical samples of *P. humuli* grouped together in this analysis, indicating no major genetic changes in this homothallic pathogen. A comparatively more complex structure was evidenced for the *P. cubensis* samples (Figure 2). Here, the European samples and the historical North American samples were placed close to one another, but the current North American samples were distinctly separated from the bulk of *P. cubensis* samples. This result was also confirmed by the dendrogram of subpopulation pairwise genetic distance matrix (Figure 2) and further suggested that the current European samples of both taxa may be direct descendants of the respective historical samples by the feature of small genetic distances. The same analysis placed the historical North American *P. cubensis* samples generally close to the bulk of the remaining ones but underlined the separation of the current North American *P. cubensis* subpopulation.

**FIGURE 3:**
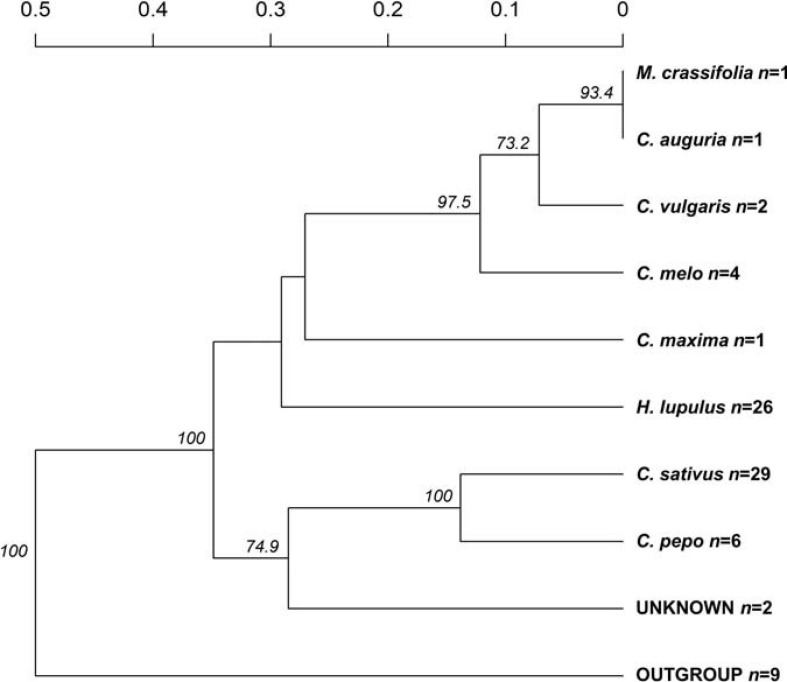
Genetic distances indicate *Pseudoperonospora cubensis*, but not *P. humuli*, host specialization. Counts for specimens analyzed from each affected host plant species are indicated next to each species nameplate (n). Bootstrap values >70% are indicated; those were calculated based on 1,000 permutations of the genotyped dataset.

The recorded information about the affected host plant species was also evaluated. Here, the relationship among nine groups of host plants was investigated (Figure 3), deriving from 70 clone-corrected MLGs. This analysis placed the *C. sativus*-based *P. cubensis* clade as the earliest and indicated divergence of the taxa and great genetic distance from the remaining samples of non-*C. sativus P. cubensis*. This bulk of non-*C. sativus* samples showed comparatively much smaller genetic distances from one another, suggesting recent or ongoing divergence within *P. cubensis*. Of note, the samples with unknown hosts were in the “*C. sativus*” group. These results agree with the Bayesian inference using STRUCTURE on the same dataset (Figure 1C).

Collectively, several lines of evidence pointed to a comparatively more pronounced population structure of *P. cubensis* than that of *P. humuli*. Genetically distinct groups were inferred by several analytical tools and positioned the current North American *P. cubensis* samples as distant to the remaining samples, with all the *P. humuli* samples placed in one genetic cluster irrespective of the analyses.

#### Demographic Analyses

Demographic investigations of a recent bottleneck were based on the “three-groups” dataset and the “species” dataset. Comparatively more signals for heterozygosity deficiencies or excesses were present in the “three-groups” dataset and within it—using the TPM. However, both models indicated a possible bottleneck signal for *P. cubensis* from Europe with heterozygosity deficiency (Supplementary Table 5). In the “species” dataset, the Wilcoxon test indicated significant heterozygosity excess for *P. cubensis*. The overall conclusion based on both mutation models across three statistical tests suggested no bottlenecks in the evolutionary recent past of either taxon (Supplementary Table 5).

**FIGURE 4:**
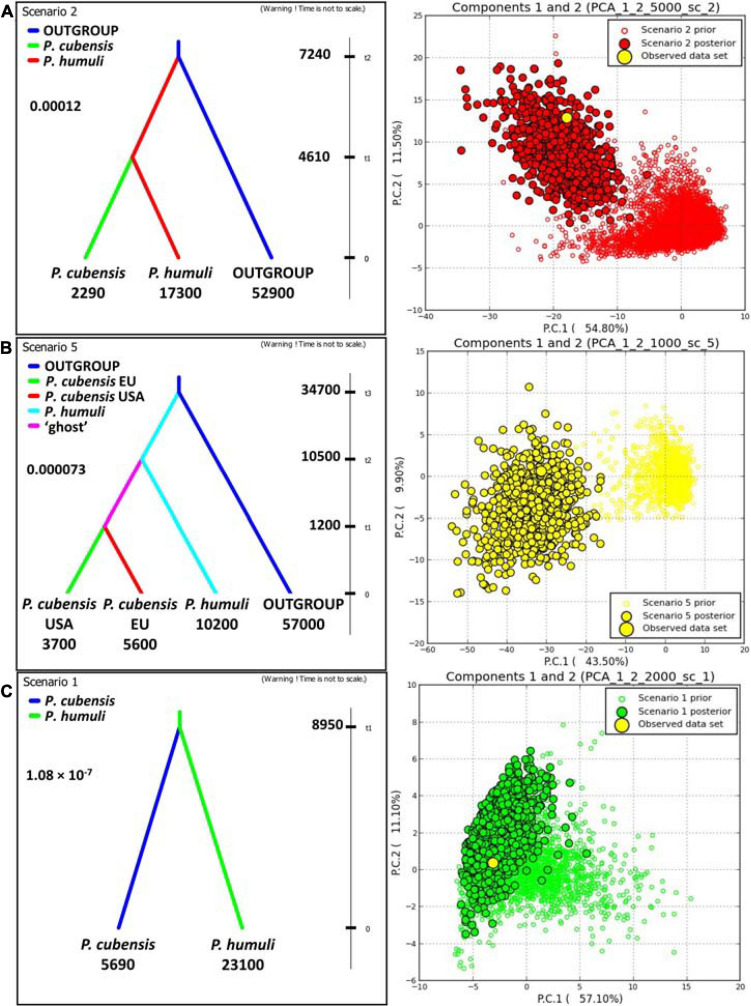
DIYABC analyses of the evolutionary relationship between *Pseudoperonospora cubensis* and *P. humuli*. Left column: best scenarios for each dataset; right column: respective model checking (PCA of priors, posteriors, and the observed dataset). **(A)** “Species” dataset with multispecific outgroup; **(B)** “three-subpopulations” dataset with multispecific outgroup; **(C)** sequence matrix across six genomic simple sequence repeats. For either analysis, 1,000,000 pseudo-observed datasets (PODs) were generated using ranges of prior indices. The subsequent analyses utilized 1% (*n* = 10,000) of the PODs closest to the observed dataset as per within- and among-subpopulation indices. The comparative analyses of both scenarios provided the support for each regarded evolutionary scenario (D, direct; L, logistic; 95% CI given). *t*_*n*_, time to split into the coalescent (generations); numbers below groups: effective population sizes; insert number: mutation rate.

Investigations of the evolutionary history using DIYABC employed both the gSSR genotyping datasets and the sequence matrices (see below; Figure 4 and Supplementary Tables 1, 7). Results from the “species” dataset were deemed as baseline for the remaining DIYABC analyses. In the gSSR “species” dataset, overwhelming support was found for scenario 2, in which *P. humuli* diverged from the ancestral downy mildews group about 7,240 generations into the coalescent and gave rise to *P. cubensis* about 4,610 generations ago. The multiple species outgroup had the largest effective population size, with *P. humuli* about one-fourth of this, and *P. cubensis* about 5% of this. The mutation rate in this dataset was about 1.2 × 10^–4^ per locus per generation (Figure 4A). Subdivision of the *P. cubensis* into samples from Europe and North America suggested that an unsampled “ghost” population gave rise to those samples (Figure 4B). Furthermore, the inferences largely agreed with those calculated for the “species” dataset (Figure 4A). Matrix of sequences from six gSSRs across 22 individuals of both species analyzed using DIYABC indicated that both taxa split about 8,950 generations ago with the effective population size of *P cubensis* about one-fourth of that inferred for *P. humuli*. The mutation rate was calculated at about 1.08 × 10^–7^ per nucleotide per generation (Figure 4C and Supplementary Table 6).

#### Sequence Comparisons

**FIGURE 5:**
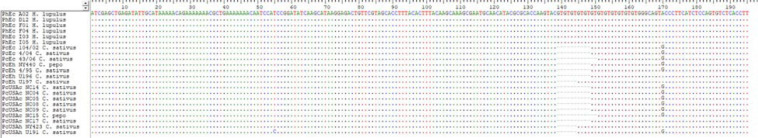
Alignment of the sequencing results for genomic simple sequence repeat (gSSR) PC021 [GT]_6_ allows clear distinction between *Pseudoperonospora cubensis* and *P. humuli*, as visualized in BioEdit (Hall, 1999). Sequence labels include preattributed subpopulation code (see Figure 1), isolate code, and the host plant identifier (Supplementary Table 1). Occasional nucleotide substitutions in the SSR flanking regions were found across all six sequenced gSSRs (Supplementary Table 7).

Analyses of the sequenced amplicons of six gSSRs from both species lent support to their distinction (Figure 5 and Supplementary Figure 5). Sequencing of the gSSR loci was limited by the availability of the pathogen gDNA, but both *Pseudoperonospora* taxa were represented by multiple samples (*n* = 22; Supplementary Table 7). Overall, the aligned gSSR sequences allowed reliable distinction between the species, with the majority of the polymorphism (insertions/deletions) located to the repeat motif regions, with occasional nucleotide substitutions in the flanking regions lending additional support for the taxon distinction (Figure 5). The obtained sequence matrix concatenated across six gSSRs was then analyzed using RAxML algorithm. The alignment of 1,049 bp had 40 patterns, with proportion of gaps and completely undetermined characters of 1.59% (this included gaps or very rare ambiguous base calls). Phylogenetic ML tree (Supplementary Figure 5) indicated only one strongly supported split, which was between *P. cubensis* and *P. humuli* samples (bootstrap of 95%).

### Comparative Genomics

The reference of 35,538 contigs of the *P. cubensis* draft genomic assembly from 2012 (Savory et al., 2012) was queried with 5,534 contigs of the *P. humuli* (Rahman et al., 2019) and showed generally high syntenic agreement with one another (Supplementary Figure 6A). Calculating the synteny between those assemblies using NUCMER revealed 11,042 contigs of *P. cubensis* were syntenic to 5,376 contigs of *P. humuli*. Thus, at the DNA level, 50.5% of the *P. cubensis* draft genome sequence was in synteny to 99.1% of the *P. humuli* draft genome. The syntenic blocks showed on average high DNA identity of 94.5 ± 4.53% (range from 50.9 to 100.0%). After six-frame translation and comparisons using PROMER, 11,301 of *P. cubensis* contigs were in synteny with 5,418 contigs of *P. humuli*. The translated syntenic blocks showed in average high amino acid identity of 88.2% (ranging from 11.6 to 92.3%) and similarity of 91.4% (ranging from 9.5 to 95.3%).

Parallel analysis using the D-GENIES pipeline indicated that more than 38% of the *P. cubensis* sequence showed nucleotide identity (>75%) when queried with the *P. humuli* contigs, whereas no match was detected in 61% of the *P. cubensis* sequence. Both genomic assemblies showed high synteny with little noise, particularly in the longer contigs of the *P. cubensis* reference (Supplementary Figure 6A, bottom-left). A large insertion in the *P. humuli* genome that was absent from the *P. cubensis* assembly was also detected in this analysis. Assemblytics-driven analyses further supported the similarities between genomes of both taxa. At the DNA level, 97% contigs of *P. humuli* mapped to 31% of the *P. cubensis* contigs, whereas after six-frame translation comparison of both assemblies, almost 98% of the *P. humuli* contigs were matched to almost 32% of the *P. cubensis* contigs. This analysis also revealed an intense tandem sequence expansion in the *P. humuli* genome, at both studied levels (Supplementary Figure 6B).

## Discussion

Here, we hypothesized that *P. cubensis* and *P. humuli*, two closely related downy mildew pathogens, are unique species. Using historical and current DNA samples of both pathogens, our results provided definitive evidence that current taxonomic separation of *P. cubensis* and *P. humuli* into separate species is valid. Our tools of choice were the *P. cubensis* gSSRs, developed shortly before the publication of the draft genome of *P. cubensis* (Kanetis et al., 2009; Savory et al., 2012).

The definition of species in the class Peronosporomycetes is complex due to the inherent underlying characteristics observed in the obligate biotrophic pathogens (Restrepo et al., 2014). Those include pseudosexual mating (noncomplete genomic shuffling), parallel asexual and sexual reproduction, and lack of segregating individuals from the hybrid, among others (Göker et al., 2007; Stukenbrock, 2013; Thines and Choi, 2016; Spring et al., 2018). Using a larger sample size than a previous work by our group (Kanetis et al., 2009), as well as available genomic resources, we were able to develop additional gSSRs that can be of great use for studies of closely related species of *Pseudoperonospora*. We were able to analyze the cross-amplification of distant/neighbor genera; however, our data pointed to limited conservation of the gSSRs within the *Pseudoperonospora* genus. Unfortunately, we did not have access to DNA samples to exhaustively test the cross-amplification due to the obligate biotrophic nature of downy mildew pathogens, but many of the currently recognized *Pseudoperonospora* species were represented in our panel. Out of the 23 confirmed gSSRs, only four cross-amplified to the related genera. Furthermore, analytical sequencing of specimens amplified with our gSSRs confirms their successful cross-transfer between both *Pseudoperonospora* taxa of focus. Here, only occasional nucleotide substitutions (but not insertions or deletions) were present in the flanking regions, whereas the bulk of the polymorphisms was detected in the repeat region of each gSSR tested this way. This feature of our gSSRs encourages their use toward pathogen detection, especially when coupling the PCR with the high-resolution melting analysis (Nowicki et al., 2015; Summers et al., 2015a; Lee et al., 2016; Rahman et al., 2019; Stackhouse, 2019). Finally, several gSSRs that successfully amplified in the initial tests of 22 specimens from both taxa across the 23 gSSRs were not found in the current draft genomes, which argues for the continued efforts toward improved genomic resources of the downy mildew pathogens.

Compared with the gSSRs used here, the recent boost in the next-generation sequencing of transcriptomes enabled accretion of the eSSRs in abundance. These eSSRs are comparatively more conserved than gSSRs, owing to the selective pressure against mutation in the coding regions of the genome, and generally capture the extant genetic diversity at a lower scale than gSSRs, as demonstrated for *P. cubensis* and closely related downy mildew pathogens (Wallace and Quesada-Ocampo, 2017). Similarly, the broadly used ISSR and RAPD markers are now considered outdated and inferior to SSRs because of the limitations inherent to either marker type, including dominant character, ambiguity in calling the null alleles, or unknown sequences targeted, even if allowed some insights into *P. cubensis* and *P. humuli* population biology (Chee et al., 2006; Polat et al., 2014).

In agreement with reports that used other methodological approaches (Göker et al., 2009; Mitchell et al., 2011; Runge et al., 2011; Runge and Thines, 2012; Summers et al., 2015b), our study strongly supported the separation of the two analyzed *Pseudoperonospora* taxa into two distinct species, based on several independent lines of evidence. These included their separation using the Bayesian inference (both STRUCTURE and DIYABC) and multivariate analysis (DAPC) of the gSSR data and ML and DIYABC analyses of the sequence data. Regarding the spatiotemporal differences in both taxa, *P. humuli* proved comparatively genetically stable, with one major cluster. This can be linked to the commonly observed sexual reproduction in this species and its homothallic character (Gent et al., 2017) and can explain the problems with chemical control strategies (Gent et al., 2008). In contrast, strong spatiotemporal differentiation was observed in *P. cubensis*, with the historical samples distinct from the current samples. The age for the collected historical samples used in this study ranged between 1887 and 2014 (*P. cubensis*) and between 1925 and 2010 (*P. humuli*). This roughly corresponds to the historical specimens of both taxa in the U.S. Fungus Database, with the oldest *P. cubensis* dated 1857 (Cuba) and *P. humuli* 1906 (Japan). Inspection of that collection and ours points that by early 20th century, *P. cubensis* was spread wherever the cucurbits were grown, and implies Europe or East Asia as the possible origin of *P. humuli* based on the frequency and age of the specimens. One further observation is, that by 1900, *P. cubensis* was virulent to crops from 10 genera of Cucurbitaceae (Supplementary Table 1, U.S. Fungus Database), which further underscores the validity of divergence we noted here. Detailed molecular analyses of those invaluable historical materials should follow to uncover the geographical origin of *P. cubensis*, best accompanied by robust population genetics/genomics of the current samples from the regions where cucurbits originate. The data corroborate the importance of mutation in generating the species diversity, and our estimates for mutation rates both at gSSR and sequence bases are well within the range for the oomycetes: *Plasmopara viticola* with 2.85 × 10^–4^ (Fontaine et al., 2013) or 1 × 10^–4^ (Gobbin et al., 2003), *Phythophthora parasitica* with 4.7 × 10^–4^ (Dunn et al., 2014), or *Peronospora tabacina* with 3.24 × 10^–4^ (Nowicki et al., 2021). One possible limitation is the exclusion of almost half of the starting collection owing to its clonal character and can be readdressed in more robust future research by using more specimens and/or more markers, which provided the desired study resolution and support for conclusions in related pathogens (Cooke and Lees, 2004; Croucher et al., 2013).

Differentiation observed in *P. cubensis* can be at least partially explained by the analyses using the host plant information. *P. cubensis s.l.* is broadly accepted as pathogenic to a wide range of Cucurbitaceae spp. hosts, and this observation led to the development of a host standard system for the virulence phenotype assessment (Sarris et al., 2009; Polat et al., 2014; Kitner et al., 2015; Spring et al., 2018). Indeed, ours and other studies (Thomas et al., 2017b; Wallace et al., 2020) support separating the taxon into distinct species, pending future developments. Furthermore, several studies leveraged this observation for important insights into the pathogen biology and epidemiology (Cohen and Rubin, 2012; Polat et al., 2014; Naegele et al., 2016; Wallace et al., 2020). For our collection, we observed the separation of the *C. sativus* specimens of *P. cubensis* from the other sampled host species, in addition to the distinct clade formed by the *H. lupulus* specimens of *P. humuli*. By using the historical specimens of both pathogens, we gained some evidence for an ongoing speciation within *P. cubensis*. Recent evidence from -omics research has added several important genomic and metabolic insights to the accumulated molecular and host–pathogen interactions and specificity evidence of oomycetes speciation (Burkhardt and Day, 2013; McGowan and Fitzpatrick, 2020; Rodenburg et al., 2020). These include alternative splicing in the effector genes (Savory et al., 2012; Summers et al., 2015b), recombination and partial reshuffling of the genetic material (Cohen and Rubin, 2012; Thomas et al., 2017b), and functionally, host jumps and geographic separation from the preferred host species, which necessitated the evolution of parasitic behavior by genetic adjustments (Dussert et al., 2019; Rahman et al., 2019; Thines, 2019; Rodenburg et al., 2020; Wallace et al., 2020). Our results for *P. cubensis* diverging from the ancestral *P. humuli* agree with the previous hypotheses in this regard based on phylogenetic analyses of several sequenced regions from both species (Mitchell et al., 2011).

Our comparative genomics analyses support the differences present in the draft genomes and add to our analyses of speciation within *Pseudoperonospora*. The syntenic comparison is overall strong, supporting the close relatedness between the two analyzed taxa. It needs to be stressed that the presently available draft assemblies cover only about 73% (*P. cubensis*) or about 54% (*P. humuli*) of their genome sizes estimated by the Feulgen method (Voglmayr and Greilhuber, 1998). This method proved reliable in assessing the genome sizes when compared with other oomycete genomes assembled and regarded as complete, such as *Albugo candida*, *B. lactucae*, or *P. viticola* (McGowan and Fitzpatrick, 2020; Rodenburg et al., 2020). We did observe a small amount of “noise” (misaligned contigs), which, as noted above, underlines the need for improved genomic resources of downy mildew pathogens. To further strengthen this point, two of our gSSRs each placed in a single contig in the *P. cubensis* assembly were present as two disjointed contigs in the *P. humuli* assembly (PC06; PC024). Similarly, PC017 allowed the identification of a 6-bp indel present in the *P. humuli* assembly. Finally, the major gap in the *P. cubensis* genome visible in the syntenic comparison indicates that a substantial part of the genomic information is still missing. This is in stark contrast to a recent robust analysis which suggested that four genes separated *P. humuli* from *P. cubensis* (Rahman et al., 2019). Notably, the draft genome of *P. humuli* reported there lent great advances to pathogen detection. Indeed, a transcriptome-based study concluded that the respective differences reach much deeper and may involve changes in expression and pathogenicity signatures across hundreds of genes (Summers et al., 2015b). Third-generation sequencing and hybrid assembly tools should help decipher the problematic gene-sparse and repeat-rich genome regions (Haas et al., 2009; McGowan and Fitzpatrick, 2020), which are the common obstacle in the effective assembly of short reads. The genotyping-by-sequencing research seems a particularly attractive analytical approach for both species, taking advantage of the comparatively small genome sizes for such a massive tool (Thomas et al., 2017a; Gent et al., 2019).

In summary, our population genetics study on the taxonomic identity of closely related downy mildew pathogens *P. cubensis* and *P. humuli* using the gSSRs provided the definite proof for the separation of both taxa. The data accrued allowed novel insights into the evolutionary history of the complex, reliably separating the *P. cubensis* samples from cucumber from those of other cucurbits, as well as *P. humuli* from hop. Our approach also allowed capturing the ongoing speciation in *P. cubensis* based on the host specificity. Leveraging the discriminating features of SSR genotyping and host information allows replacement of the current resource-intensive bioassay used for pathotyping of *P. cubensis* isolates. Characterizing *P. cubensis* in the regions where the cucurbits originate could help prove our inference of species origin. We also observed the need for improved genomic resources for those important pathogens, which can further inform the species biology and direct the development of related control measures.

## Data Availability Statement

The datasets presented in this study can be found in the article/Supplementary Material.

## Author Contributions

MN: data generation, data analysis, primary writing, and editing. DH: data generation, data analysis, writing, and revisions. RT: project idea, financial support, project oversight, resources, data generation, writing, and revisions. SB: data generation, data management, project management, writing, and revisions. LK and PW: data generation, writing, and revisions. PO, MC, OS, MT, FR, and BS: data generation, resources, writing, and revisions. All authors contributed to the article and approved the submitted version.

## Conflict of Interest

The authors declare that the research was conducted in the absence of any commercial or financial relationships that could be construed as a potential conflict of interest.

## Supplementary Material

Supplementary Figure 1 Genotype accumulation curves for *Pseudoperonospora cubensis* and *P. humuli* “6-subpopulations” datasets.

Supplementary Figure 2 Hardy-Weinberg equilibrium (HWE) violations for “species” **(A)** and “6-subpopulations” datasets of *Pseudoperonospora cubensis* and *P. humuli* **(B)**. Color scale indicates high (blue) and low (pink) support for HWE violations across loci (rows) and groups analyzed (columns).

Supplementary Figure 3 Pairwise linkage disequilibrium among the genomic simple sequence repeats analyzed, standardized by the population size in *Pseudoperonospora cubensis* and *P. humuli* populations.

Supplementary Figure 4 Isolation-by-Distance analysis (Mantel and partial Mantel tests) for *Pseudoperonospora cubensis* **(A,B)** and *P. humuli* **(C,B)**. Correlation between matrices of genetic distances (vertical axis, Dgen) and geographical distances (horizontal axis, Dgeo) **(A,C)** were calculated using raw data (*r*) or standardized by year of sample (*r’*). Significance of the result was calculated by 999 permutations of the dataset. Correlograms for either species [**(C,D)** respectively) indicate the Mantel’s *r* for each given geographical distance class, with significant results (*P* < 0.05) marked with filled symbols.

Supplementary Figure 5 Maximum Likelihood tree of the concatenated and MAFFT-aligned sequences of six genomic simple sequence repeats amplified using gDNA samples of *Pseudoperonospora cubensis* (*n* = 14) and *P. humuli* (*n* = 3). Not shown: clonal sequences (*n* = 5; 3 for *P. cubensis* and 2 for *P. humuli*) removed prior to running the RAxML with deep bootstrap. Bootstrap support values for each split are indicated using the color legend. Edge labels include sample identifier (Supplementary Tables 1, 7) and pre-attributed group identifier that reflected the sample geographic origin and age (see Figure 1).

Supplementary Figure 6 Comparison of the genomic assemblies of *Pseudoperonospora cubensis* (reference) and *P. humuli* (query). **(A)** Visualization of the syntenic comparison of the draft genomes of both taxa, using D-genies and minimap2 algorithm. Organisms and genomes are identified along the respective axes. The cumulative sizes of the compared sequences are also indicated, respectively. The reference *P. cubensis* contigs were arranged in the decreasing size order along the horizontal axis. **(B)** Structural variants in the *P. cubensis* (reference; left direction from the middle) and *P. humuli* (query; right direction from the middle) draft genomes visualized with Assemblytics based on the delta files from the global alignments using MUMMER at DNA level (NUCMER; top panel) and at protein level after 6-frame translation (PROMER; bottom panel). Variant types are color-coded according to the legend in the insert.

Supplementary Table 1 Complete binned genotyping dataset used for this study. The table identifies the sample by its original code, population code (“6-subpopulation” manner), detected alleles per each tested locus, state/country of origin, year of collection, and affected host plant species. Additional formatting was put for the samples used in the initial testing of the 27 genomic simple sequence repeats (gSSRs) (bold), and those used for sequencing across selected 6 gSSRs (highlight).

Supplementary Table 2 Genomic simple sequence repeat details, including the internal code (PC#), the repeated motif, forward and reverse primers, expected size length, genomic locations to *P. cubensis* and *P. humuli* draft genomes (if detected; contig and bases within identified), PCR product size range in the initial study (Supplementary Table 1), and results of cross-amplification to related Oomycetes species (PCR product sizes only if amplified).

Supplementary Table 3 Results of the analysis of molecular variance (AMOVA) test for variance partitioning (“6-subpopulations”, “species”, and “host” datasets).

Supplementary Table 4 Summary of ObStruct analyses of the STRUCTURE outcome. For each performed analysis (“6-subpopulations”, “species”, “host” datasets), the resultant files for 30 MCMCs were pooled, to indicate how the fit statistics *R*^2^ changed, when sequentially removing the pre-attributed populations or the inferred clusters.

Supplementary Table 5 Results of the Bottleneck analyses (“3-groups” and “species” datasets). Bottleneck used the stepwise mutation model (S.M.M.) and the twho-phase mutational model (T.P.M.), to test for shift in the inferred population counts. Significance of heterozygosity excess/deficiency for each group and mutation model was also assessed. Conclusion about shifts in the population size (“mode shift” descriptor) takes into consideration all three basic tests (sign, standardized differences, and Wilcoxon sign-rank tests).

Supplementary Table 6 Results of the DIYABC analyses (“3-groups” and “species” datasets). For the best-supported scenario for each analysis, the estimates for effective population sizes, mutation rates (μ) and times of split (t_*x*_) are indicated as medians and respective 95% CI ranges.

Supplementary Table 7 Details of the analytical sequencing of 22 gDNA samples of both taxa across 6 selected genomic simple sequence repeats (gSSRs). Affected host plants, consensus sequences, and the respective GenBank submission numbers are indicated.
