# Erratum: “Jumping Jack”: Genomic Microsatellites Underscore the Distinctiveness of Closely Related *Pseudoperonospora cubensis* and *Pseudoperonospora humuli* and Provide New Insights Into Their Evolutionary Past

**DOI:** 10.3389/fmicb.2021.772633

**Published:** 2021-09-29

**Authors:** Frontiers Production Office

**Affiliations:** Frontiers Media SA, Lausanne, Switzerland

**Keywords:** oomycete, obligate pathogens, downy mildew, speciation, evolution, genotyping, host specificity

Due to a production error, Supplementary Data Sheet 1 was mistakenly not published. The publisher apologizes for this mistake. The original article has been updated.

