# Supplementary material for: “Jumping Jack”: Genomic Microsatellites Underscore the Distinctiveness of Closely Related Pseudoperonospora cubensis and Pseudoperonospora humuli and Provide New Insights Into Their Evolutionary Past
Source: Front Microbiol. 2021 Jul 14;12:686759. doi: 10.3389/fmicb.2021.686759 (PMC8317435; doi:10.3389/fmicb.2021.686759)
Supplement: Supplementary Figure 1 — Genotype accumulation curves for Pseudoperonospora cubensis and P. humuli “6-subpopulations” datasets. [file Data_Sheet_1.docx]

**Supplementary Figures SF1 to SF6**


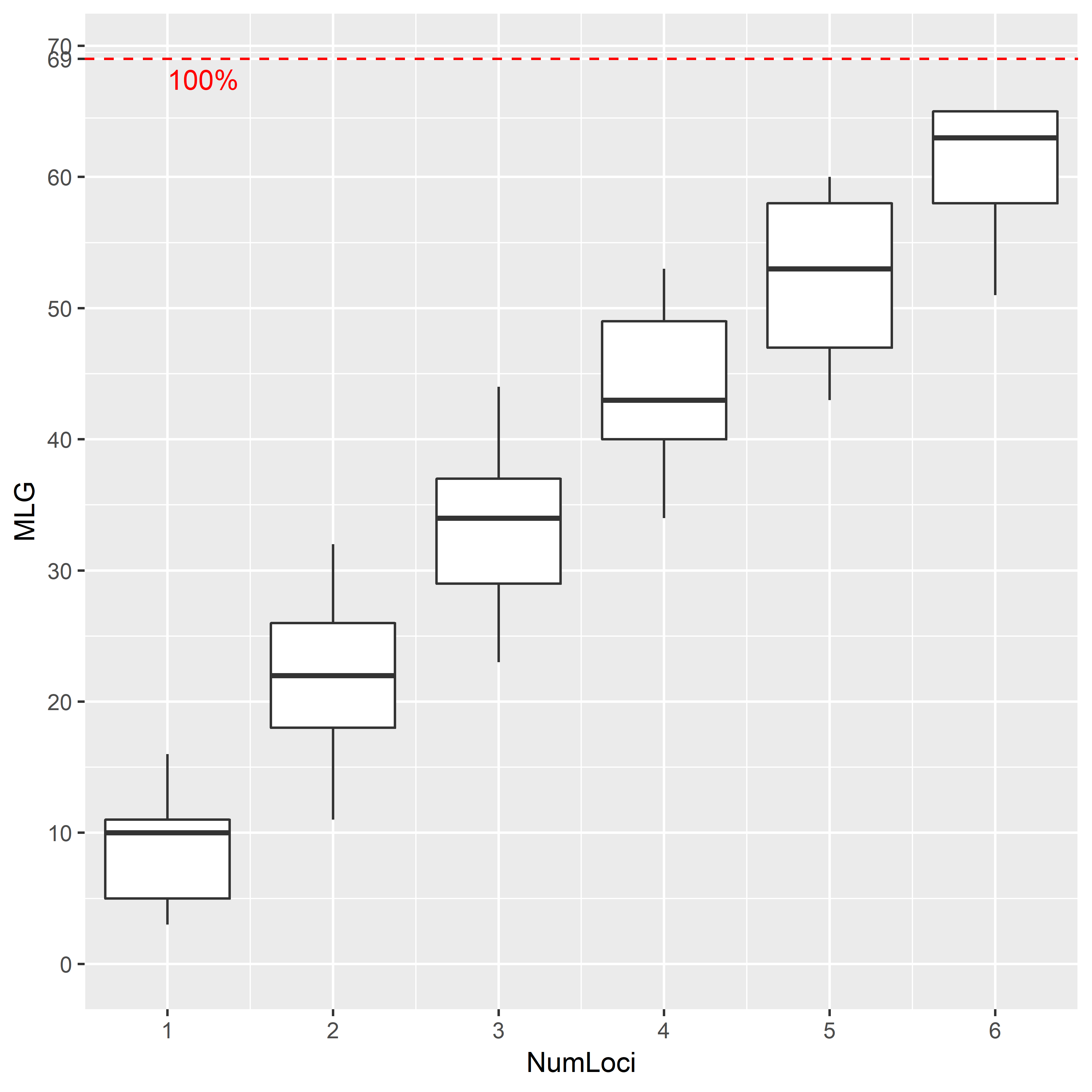


Supplementary Fig. F1: Genotype accumulation curves for *Pseudoperonospora cubensis* and *P. humuli* ‘6-subpopulations’ datasets.


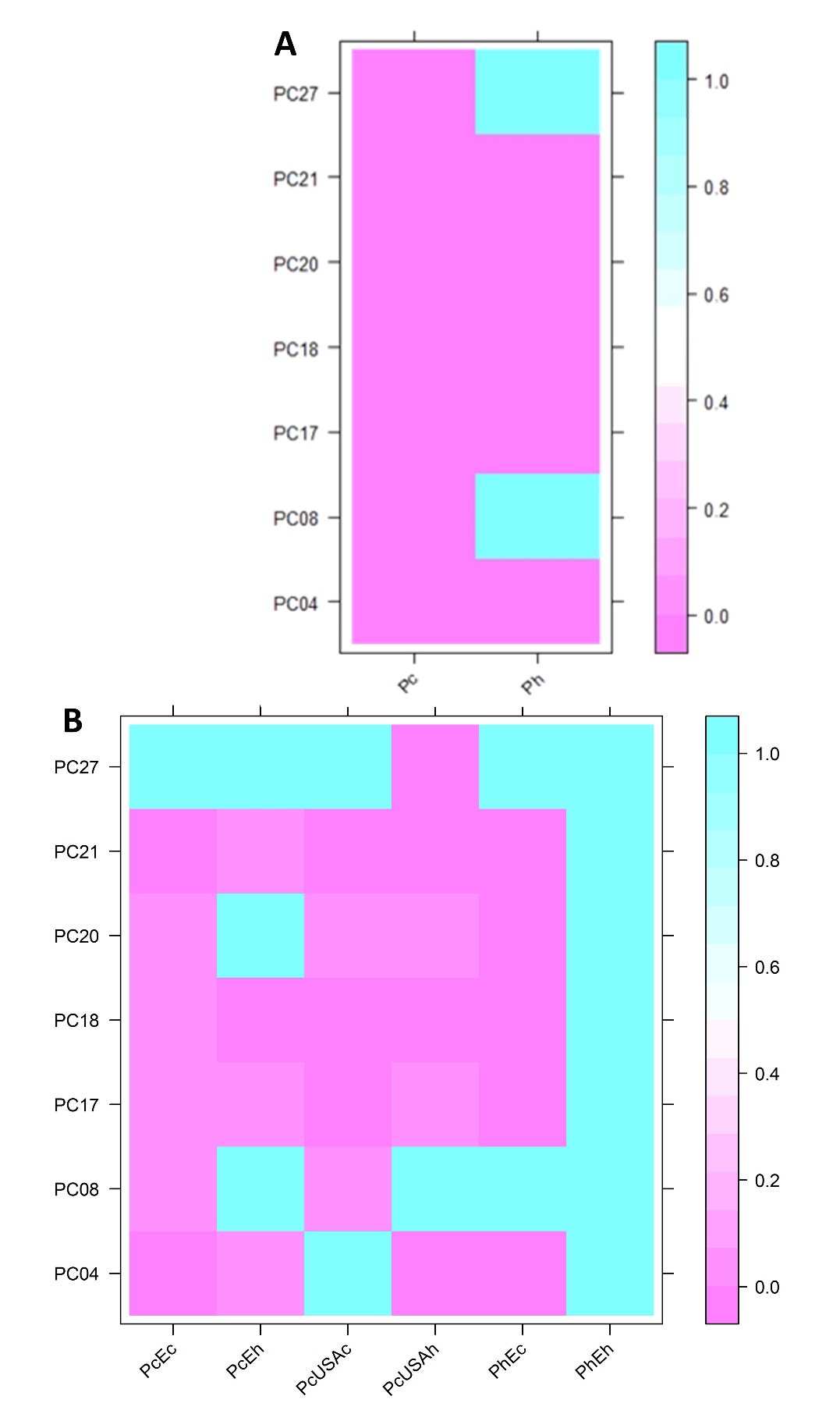


Suppl. Fig. F2: Hardy-Weinberg equilibrium (HWE) violations for ‘species’ (A) and ‘6-subpopulations’ datasets of *Pseudoperonospora cubensis* and *P. humuli* (B). Color scale indicates high (blue) and low (pink) support for HWE violations across loci (rows) and groups analyzed (columns).


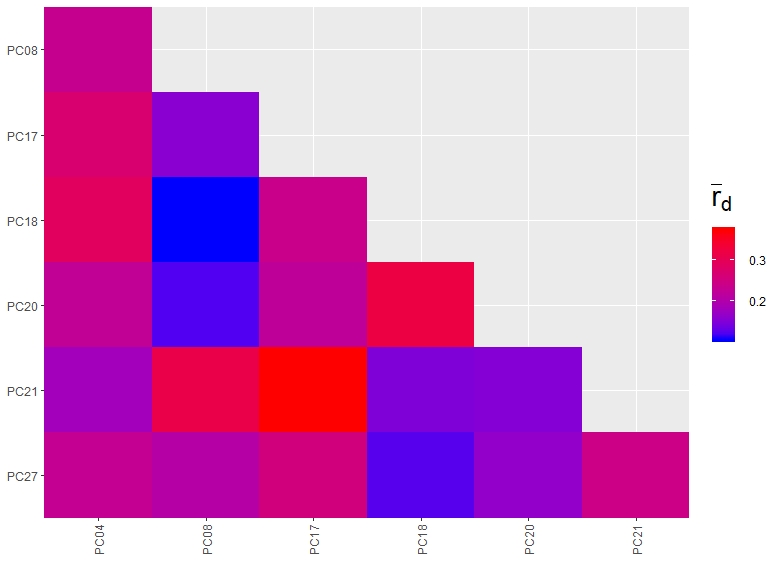


Suppl. Fig. F3: Pairwise linkage disequilibrium among the genomic simple sequence repeats analyzed, standardized by the population size in *Pseudoperonospora cubensis* and *P. humuli* populations.


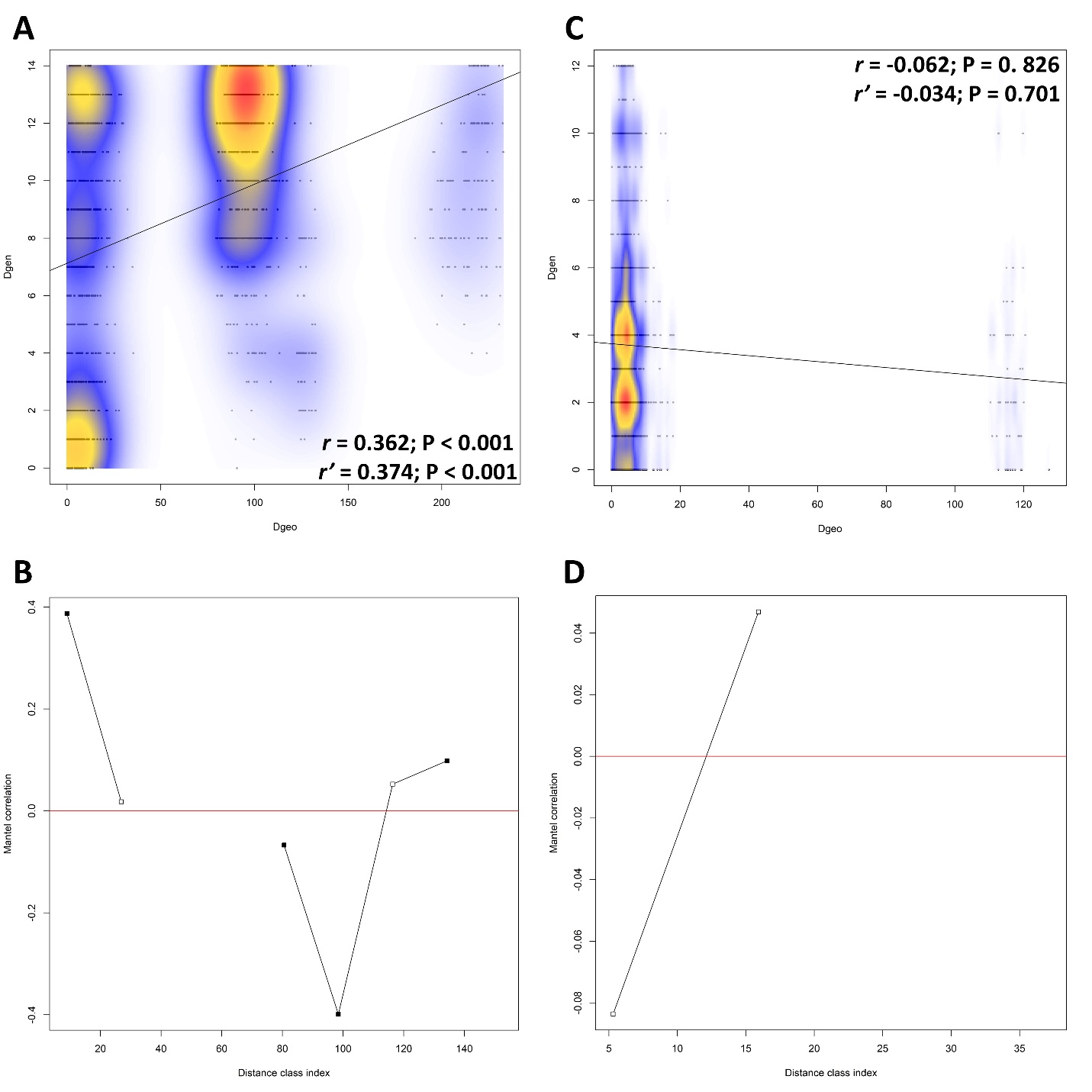


Suppl. Fig. F4. Isolation-by-Distance analysis (Mantel and partial Mantel tests) for *Pseudoperonospora cubensis* (A,B) and *P. humuli* (C,B). Correlation between matrices of genetic distances (vertical axis, Dgen) and geographical distances (horizontal axis, Dgeo) (A,C) were calculated using raw data (*r*) or standardized by year of sample (*r’*). Significance of the result was calculated by 999 permutations of the dataset. Correlograms for either species (C,D, respectively) indicate the Mantel’s *r* for each given geographical distance class, with significant results (P <0.05) marked with filled symbols.


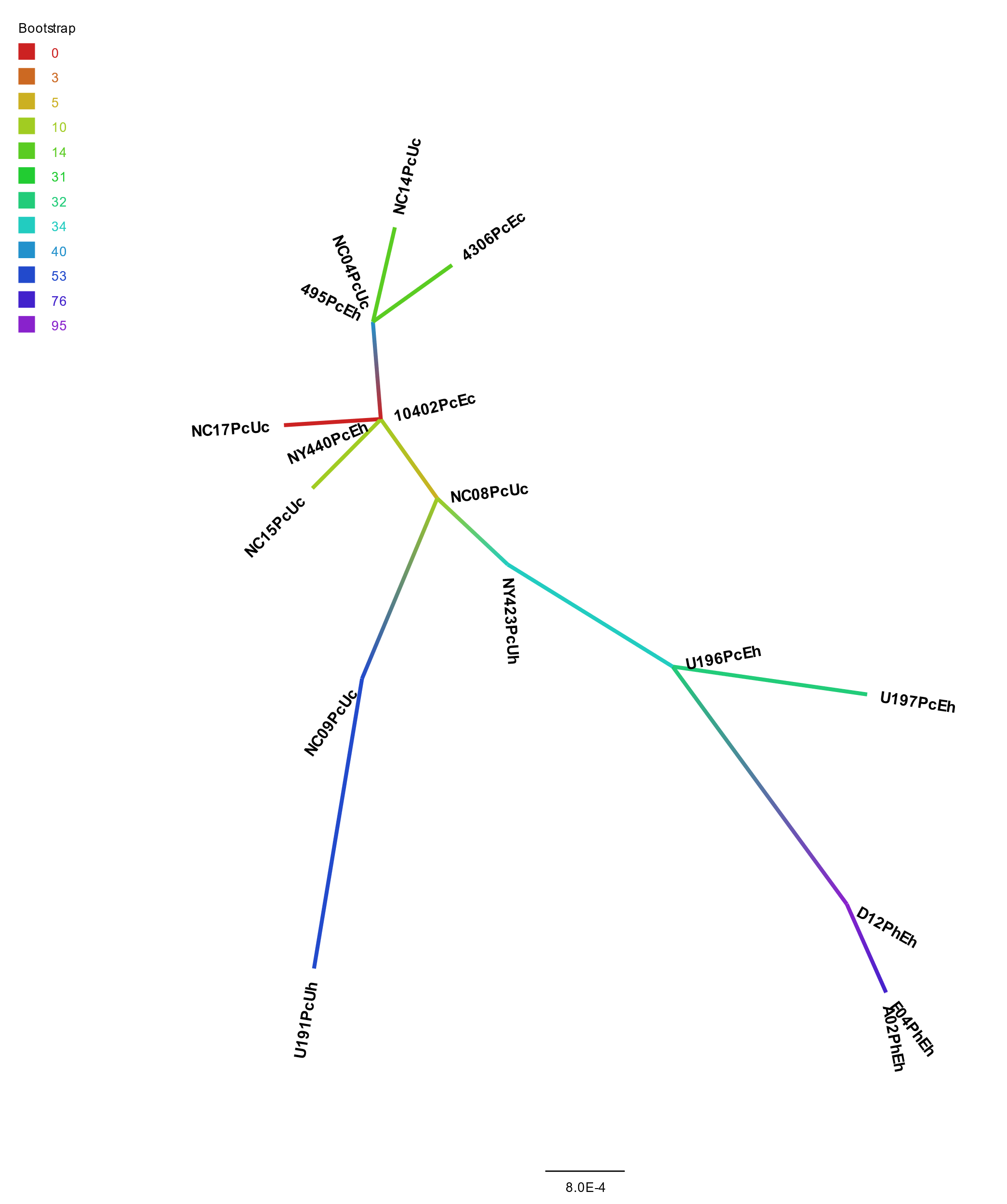


Suppl. Fig. F5. Maximum Likelihood tree of the concatenated and MAFFT-aligned sequences of six genomic simple sequence repeats amplified using gDNA samples of *Pseudoperonospora cubensis* (*n* = 14) and *P. humuli* (*n* = 3). Not shown: clonal sequences (*n* = 5; 3 for *P. cubensis* and 2 for *P. humuli*) removed prior to running the RAxML with deep bootstrap. Bootstrap support values for each split are indicated using the color legend. Edge labels include sample identifier (Suppl.Tables T1 and T7) and pre-attributed group identifier that reflected the sample geographic origin and age (see Fig. 1).


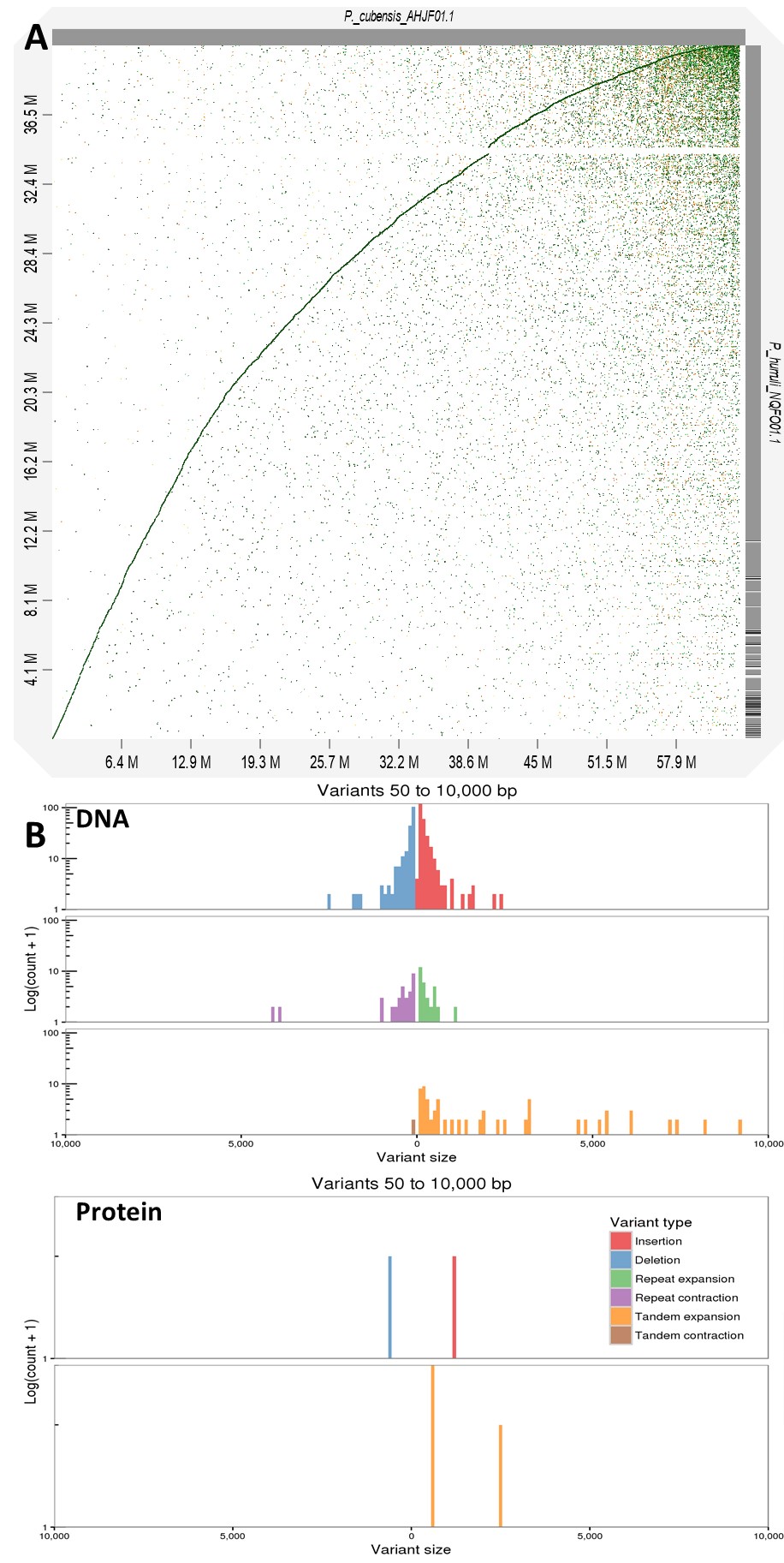


Suppl. Fig. F6. Comparison of the genomic assemblies of *Pseudoperonospora cubensis* (reference) and *P. humuli* (query). (A) Visualization of the syntenic comparison of the draft genomes of both taxa, using D-genies and minimap2 algorithm. Organisms and genomes are identified along the respective axes. The cumulative sizes of the compared sequences are also indicated, respectively. The reference *P. cubensis* contigs were arranged in the decreasing size order along the horizontal axis. (B) Structural variants in the *P. cubensis* (reference; left direction from the middle) and *P. humuli* (query; right direction from the middle) draft genomes visualized with Assemblytics based on the delta files from the global alignments using MUMMER at DNA level (NUCMER; top panel) and at protein level after 6-frame translation (PROMER; bottom panel). Variant types are color-coded according to the legend in the insert.
