# Supplementary material for: “Jumping Jack”: Genomic Microsatellites Underscore the Distinctiveness of Closely Related Pseudoperonospora cubensis and Pseudoperonospora humuli and Provide New Insights Into Their Evolutionary Past
Source: Front Microbiol. 2021 Jul 14;12:686759. doi: 10.3389/fmicb.2021.686759 (PMC8317435; doi:10.3389/fmicb.2021.686759)
Supplement: Supplementary Figure 2 — Hardy-Weinberg equilibrium (HWE) violations for “species” (A) and “6-subpopulations” datasets of Pseudoperonospora cubensis and P. humuli (B). Color scale indicates high (blue) and low (pink) support for HWE violations across loci (rows) and groups analyzed (columns). [file Data_Sheet_2.docx]

**Supplementary File SF1: Method description**

Description of DIYABC method and results.

Evolutionary history of the species was investigated using the approximate Bayesian computation using the DIYABC version 2.1 (Cornuet *et al.*, 2014). The focus of these analyses was on inferring the evolutionary relationships among both *Pseudoperonospora* species in relation to other downy mildew pathogens genotyped using the same seven gSSRs (Suppl. Table T1, Suppl. Table T2). The initial run established the limits of the input indices (population sizes, mutation rates) and used the whole dataset as one population, with the parameters stretched one order of magnitude beyond those defaulted by the program. The subsequent full DIYABC analyses were only run using the pre-calculated 95% confidence interval (CI-95%) values for those indices using the ‘species’ dataset (Outgroup, *Pc*, *Ph*) dataset and ‘3-subpopulations’ dataset (Outgroup, *Pc* from Europe, *Pc* from USA, *Ph*). Specimens used for multispecific Outgroup are listed out in the Suppl. Table T1 and T2.

Two-tiered approach was used for DIYABC. In the initial run, the dataset was treated as one population, and focused on establishing limits of the input indices (population sizes, mutation rates), with the parameters stretched one order of magnitude beyond those defaulted by the program, with assumptions of S.M.M. mutation model. The subsequent full DIYABC analysis was only run using the pre-calculated 95% confidence interval (CI-95%) values for those indices for both dataset subdivisions. Pooling of groups of samples with shared histories/locations reduces the complexity of the evaluated scenarios and is frequently used for the DIYABC approach (Cornuet et al., 2014, Jeon, 2018, Kim *et al.*, 2019, Zhang *et al.*, 2019). The ‘species’ genotyping dataset was tested with 3 possible evolutionary scenarios that considered sequential divergence or a ‘ghost’ (unsampled) originating population (Fig. 1 below). The ‘3-subpopulations’ dataset was tested with 5 possible evolutionary scenarios that considered sequential divergence or a ‘ghost’ (unsampled) originating population (Fig. 2 below).

DIYABC generated a reference table of a 1,000,000 pseudo-observed datasets (PODs) under the assumed generalized S.M.M. with a uniform distribution of mutation rate from 5.44 × 10^-4^ to 9.01 × 10^-4^ per locus and per generation. Population size minimum of 547 and maximum of 779000 were used, with split times between 10 and 100,000 generations into the coalescent, all parameters under uniform distribution.

The sequence matrix dataset (only *P. cubensis* and *P. humuli*) was tested with 2 possible evolutionary scenarios that considered a divergence from *P. humuli* or a ‘ghost’ (unsampled) originating population (Fig. 3 below). This dataset was tested under the assumed Kimura2 mutation model (Kimura, 1980) with a uniform distribution of mean mutation rate from 3.29 × 10^-8^ to 1.56 × 10^-7^ per nucleotide and per generation, or individual locus mutation rate range between 2.31 × 10^-8^ to 9.10 × 10^-7^ per nucleotide and per generation. Population size minimum of 2110 and maximum of 89400 were used, with split times between 10 and 100,000 generations into the coalescent, all parameters under uniform distribution.

Similarity of actual and simulated datasets was assessed using the mean number of alleles, mean genetic diversity and mean size variance for single population, and pairwise F_ST_, classification index, and (dμ)^2^ distance for pairs of populations. Principal components analysis (PCA) pre‐evaluation step was also performed to ensure that at least one combination of scenarios and priors produced PODs close enough to the observed data set. The relative posterior probabilities of both scenarios undergoing comparison were inferred by the direct (D) and logistic (L) regression on the 1% of PODs closest to the observed data set (Cornuet *et al.*, 2010). A model with the highest relative posterior probability between those considered, and for which the CI-95% did not overlap with the CIs of the other models, was considered the best model. We also fed the results of the bigger-scale analyses into more detailed runs, with the ‘species’ dataset informing all subsequent analyses. Model checking algorithm was also applied to evaluate the goodness-of-fit of the chosen best scenario using PCA, in which the observations were the PODs (both priors and 1% posteriors closest to observed dataset) and the variables were the summary statistics listed out above. In the comparative analysis of these two scenarios, the “Bias and precision” analysis was done; also “Confidence in scenario choice” analysis was carried-out to estimate the type I and type II errors based on 1,000 PODs. Small type II errors provided good confidence in the selected scenario even if the type I errors were large.


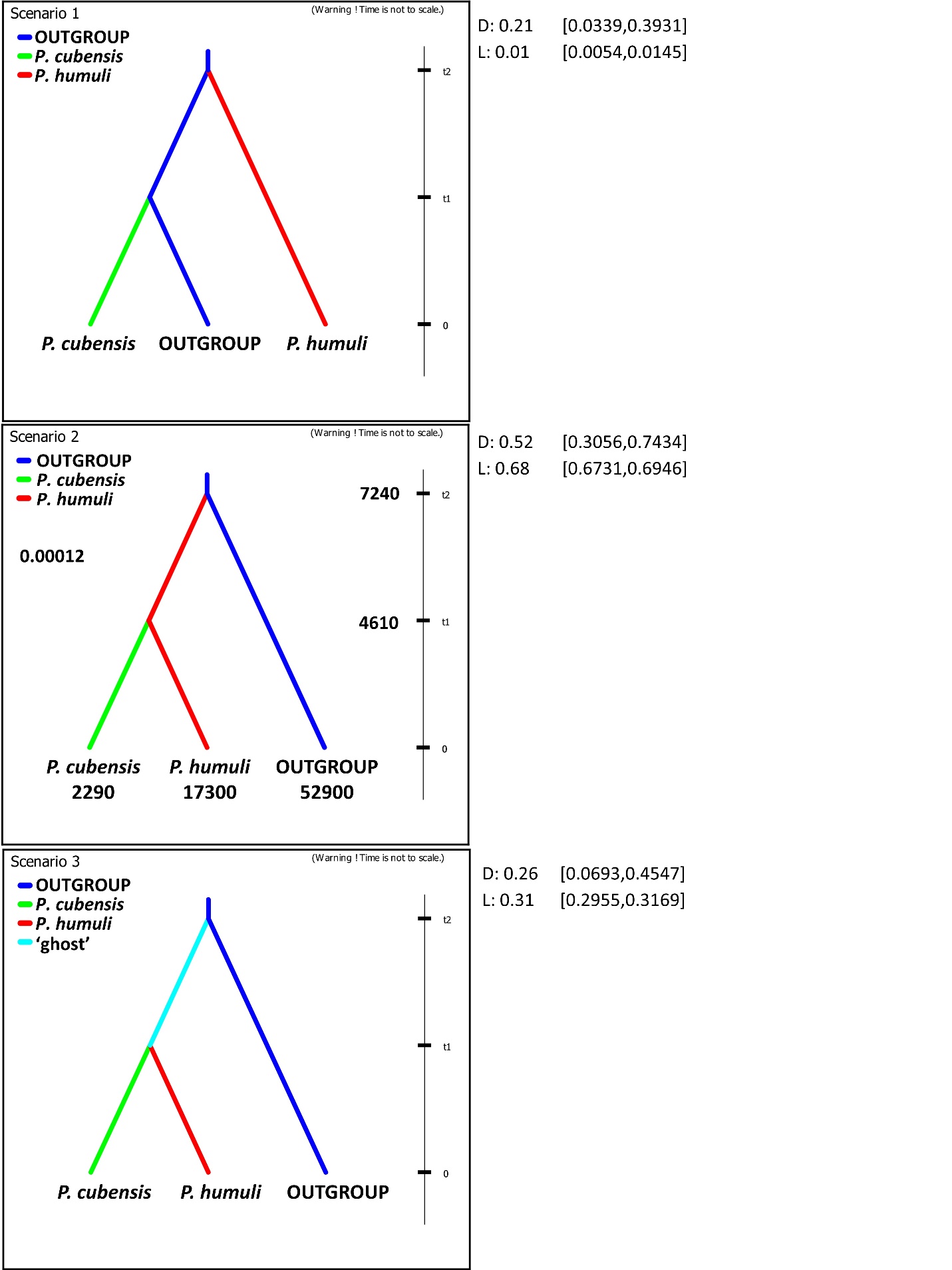


Figure 1. Evolutionary scenarios considered for the ‘species’ dataset. Multispecific Outgroup included representative of downy mildew pathogens (Suppl. Table T1 and T2). The DIYABC analysis assessed the clone corrected data of 138 *Pseudoperonospora cubensis* and *P. humuli* multi locus genotypes using seven microsatellite loci. For either regarded scenario, 1,000,000 pseudo-observed datasets (PODs) were generated using the prior indices ranges. The subsequent analyses utilized 1% (n = 10,000) of the PODs closest to the observed dataset as per the within and among sub-population indices. The comparative analyses of both scenarios provided the support for each regarded evolutionary scenario (**D:** direct; **L:** logistic; 95% CI given). t_n_ - time to split into the coalescent [generations]; Numbers below groups: Effective population sizes; Insert number: mutation rate. Details provided only for the scenario with the highest *post-hoc* support.


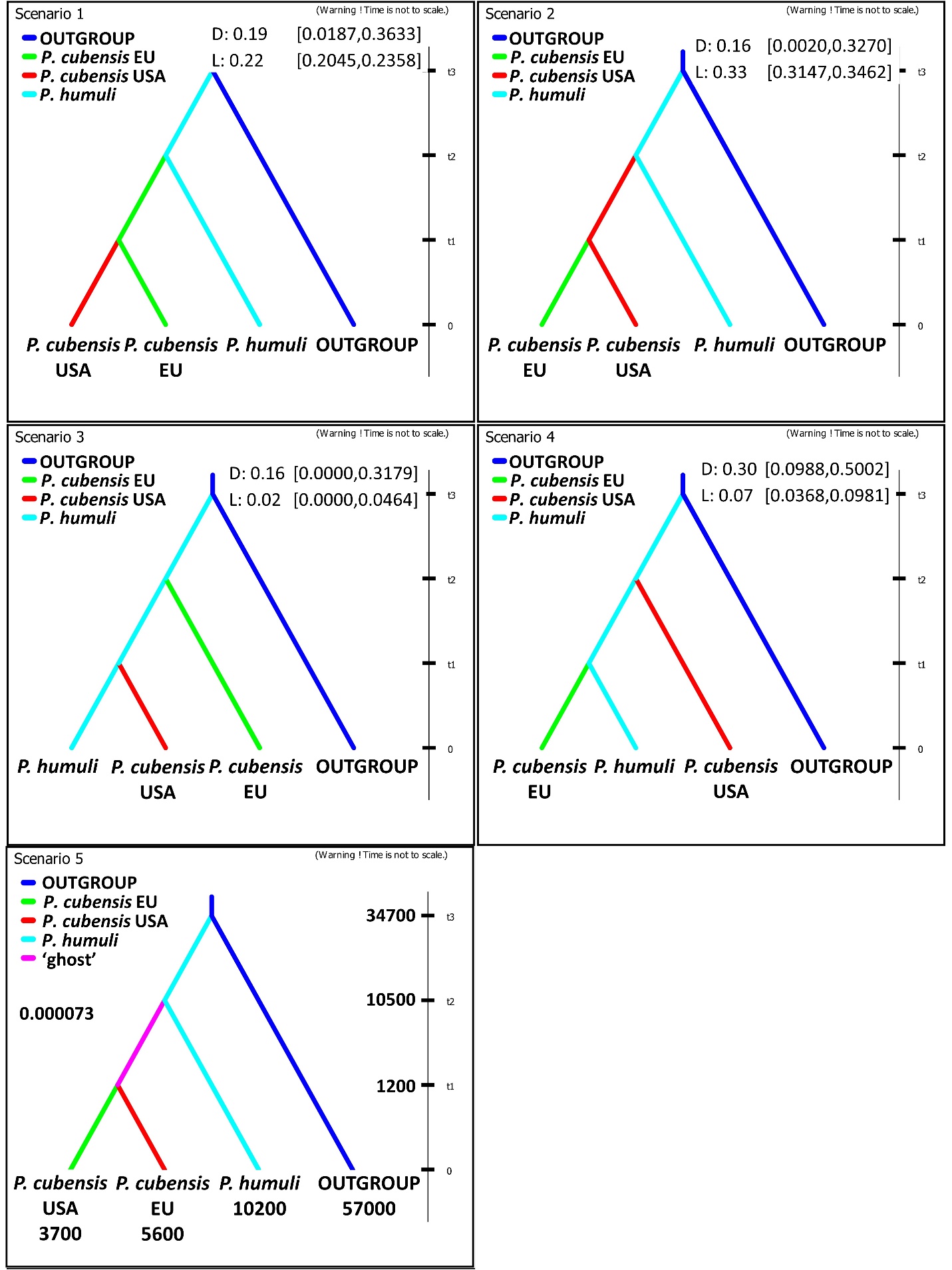


Figure 2. Evolutionary scenarios considered for the ‘3-subpopulations’ dataset. Multispecific Outgroup included representative of downy mildew pathogens (Suppl. Table T1 and T2). The DIYABC analysis assessed the clone corrected data of 138 *Pseudoperonospora cubensis* (samples from EU: Europe; USA: United States of America) and *P. humuli* multi locus genotypes using seven microsatellite loci. For either regarded scenario, 1,000,000 pseudo-observed datasets (PODs) were generated using the prior indices ranges. The subsequent analyses utilized 1% (n = 10,000) of the PODs closest to the observed dataset as per the within and among sub-population indices. The comparative analyses of both scenarios provided the support for each regarded evolutionary scenario (**D:** direct; **L:** logistic; 95% CI given). t_n_ - time to split into the coalescent [generations]; Numbers below groups: Effective population sizes; Insert number: mutation rate. Details provided only for the scenario with the highest *post-hoc* support.


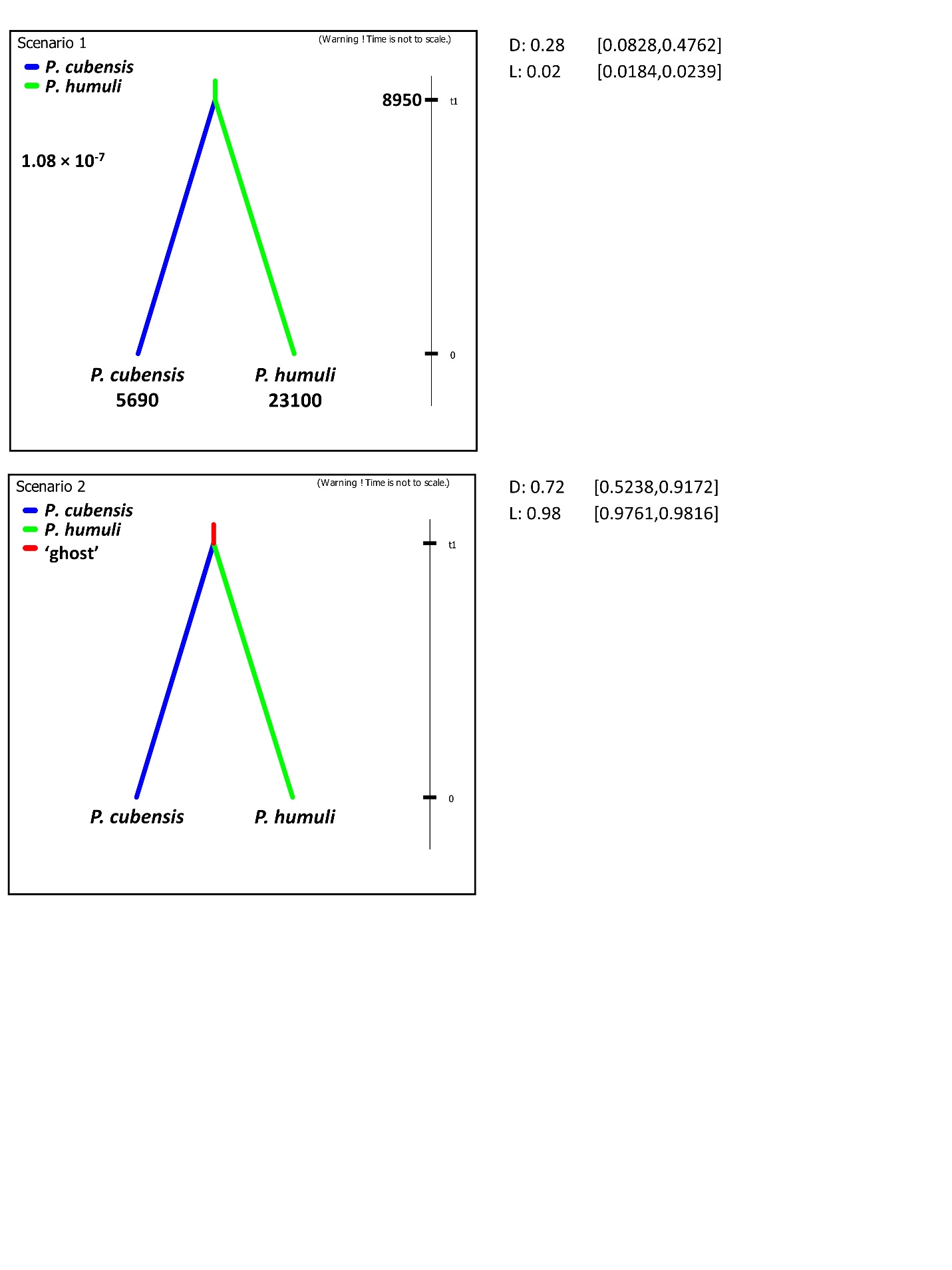


Figure 3. Evolutionary scenarios considered for the sequence matrix dataset. The DIYABC analysis assessed the clone corrected data of 22 *Pseudoperonospora cubensis* and *P. humuli* sequenced across six microsatellite loci. For either regarded scenario, 1,000,000 pseudo-observed datasets (PODs) were generated using the prior indices ranges. The subsequent analyses utilized 1% (n = 10,000) of the PODs closest to the observed dataset as per the within and among sub-population indices. The comparative analyses of both scenarios provided the support for each regarded evolutionary scenario (**D:** direct; **L:** logistic; 95% CI given). t_n_ - time to split into the coalescent [generations]; Numbers below groups: Effective population sizes; Insert number: mutation rate. Details provided only for the scenario with the highest *post-hoc* support.

References:

Cornuet, J.-M., Pudlo, P., Veyssier, J., Dehne-Garcia, A., Gautier, M., Leblois, R.*, et al.* (2014) DIYABC v2. 0: a software to make approximate Bayesian computation inferences about population history using single nucleotide polymorphism, DNA sequence and microsatellite data. *Bioinformatics,* **30,** 1187-1189.

Cornuet, J.-M., Ravigné, V. and Estoup, A. (2010) Inference on population history and model checking using DNA sequence and microsatellite data with the software DIYABC (v1. 0). *BMC Bioinformatics,* **11,** 401.

Jeon, H.-B. (2018) Evolutionary history of acheilognathid fish inferred based on genetic and genomic analyses. In: *Life Sciences.* Gyeongsan, North Gyeongsang, South Korea: Yeungnam University, pp. 377.

Kim, J., Ni, G., Kim, T., Chun, J. Y., Kern, E. M. and Park, J. K. (2019) Phylogeography of the highly invasive sugar beet nematode, *Heterodera schachtii* (Schmidt, 1871), based on microsatellites. *Evolutionary applications,* **12,** 324-336.

Kimura, M. (1980) A simple method for estimating evolutionary rates of base substitutions through comparative studies of nucleotide sequences. *Journal of Molecular Evolution,* **16,** 111-120.

Zhang, J., Yao, J., Hu, Z. M., Jueterbock, A., Yotsukura, N., Krupnova, T. N.*, et al.* (2019) Phylogeographic diversification and postglacial range dynamics shed light on the conservation of the kelp *Saccharina japonica*. *Evolutionary Applications***,** 1-13.
